# Subgingival microbiota composition is associated with brain health in the general population–the PAROMIND study

**DOI:** 10.1016/j.ebiom.2026.106312

**Published:** 2026-05-30

**Authors:** Marvin Petersen, Carolin Walther, Katrin Borof, Guido Heydecke, Thomas Beikler, Malik Alawi, Christian Müller, Felix L. Nägele, Birgit-Christiane Zyriax, Jens Fiehler, Jürgen Gallinat, Simone Kühn, Raphael Twerenbold, Corinna Bang, Götz Thomalla, Bastian Cheng, Ghazal Aarabi

**Affiliations:** a Department of Neurology, University Medical Centre Hamburg-Eppendorf, Hamburg, Germany; b Department of Periodontics, Preventive and Restorative Dentistry, University Medical Center Hamburg-Eppendorf, Hamburg, Germany; c Department of Prosthetic Dentistry, University Medical Center Hamburg-Eppendorf, Hamburg, Germany; d Bioinformatics Core, University Medical Center Hamburg-Eppendorf, Hamburg, Germany; e Midwifery Science-Health Services Research and Prevention, Institute for Health Services Research in Dermatology and Nursing (IVDP), University Medical Center Hamburg-Eppendorf, Hamburg, Germany; f Department of Neuroradiology, University Medical Center Hamburg-Eppendorf, Hamburg, Germany; g Department of Psychiatry and Psychotherapy, University Medical Center Hamburg-Eppendorf, Hamburg, Germany; h Lise Meitner Group for Environmental Neuroscience, Max Planck Institute for Human Development, Berlin, Germany; i Department of Cardiology, University Heart and Vascular Center, Hamburg, Germany; j Epidemiological Study Center, University Medical Center Hamburg-Eppendorf, Hamburg, Germany; k German Center for Cardiovascular Research (DZHK), Partner Site Hamburg/Kiel/Luebeck, Hamburg, Germany; l University Center of Cardiovascular Science, University Heart and Vascular Center, Hamburg, Germany; m Institute of Clinical Molecular Biology, Kiel University, Kiel, Germany

**Keywords:** Oral microbiome, Periodontitis, Oral-brain axis, Brain health, Cognition, Neuroimaging

## Abstract

**Background:**

Periodontitis has gained attention as a key factor associated with cognitive decline and Alzheimer's dementia. However, the relationship between periodontitis-related oral microbiota shifts and brain health in the general population remains unclear.

**Methods:**

We investigated the oral microbiome–brain axis in 1026 participants from the population-based PAROMIND Study. Using 16S rRNA gene amplicon sequencing of subgingival crevicular fluid, we inferred via topological data analysis a microbiota similarity network. This network, which distills the complex high-dimensional data into an interpretable map of microbial similarity, revealed a continuous disease gradient mirroring the microbial pathogenicity spectrum, from taxa of low periodontal pathogenicity (e.g., *Streptococcus*) to periodontitis-associated taxa (e.g., *Porphyromonas, Fusobacterium*). Leveraging this network, we systematically examined associations between periodontal microbiota profiles and 40 brain health-related phenotypes, including cognition, brain structure, mental health, inflammatory biomarkers, diet, vascular risk factors, and demographics.

**Findings:**

Higher abundance of periodontitis-related bacterial taxa was associated with poorer cognitive performance, elevated leucocyte counts, and lower MIND diet adherence after covariate adjustment. Complementary forward model selection analysis supported the links to cognitive performance and inflammation, and additionally identified a significant association with brain structure (cortical thickness and subcortical volume). We identified associations with both established genera (*Porphyromonas*) and taxa not previously implicated in brain health (*Fretibacterium, Tannerella, Dialister*).

**Interpretation:**

These findings from a large cohort advance the understanding of the oral microbiome–brain axis, highlighting specific microbial profiles linked to subclinical cognitive, structural, and inflammatory brain health markers. By demonstrating these links in a non-demented population, our study suggests that monitoring the oral microbiome could inform early risk assessment for cognitive decline, positioning periodontal health as an accessible target for early intervention strategies.

**Funding:**

Deutsche Forschungsgemeinschaft.

**Research in context**

**Evidence before this study**

Previous epidemiological and clinical studies have linked periodontitis to cognitive decline and Alzheimer's disease, and experimental work suggests that periodontal pathogens may promote systemic inflammation and neurodegenerative processes. However, most human microbiome studies have focused on patients with established dementia, used small samples, or lacked comprehensive brain imaging and cognitive testing data. Evidence from large population-based cohorts integrating subgingival microbiota profiles with multidimensional brain health measures has been limited.

**Added value of this study**

This study provides population-based evidence that variation in subgingival oral microbiota composition is associated with cognitive performance, systemic inflammation, diet, and brain structure in non-demented adults. Using a topology-based analytical framework, we identify a continuous periodontitis-related microbial gradient and link both known and previously unreported bacterial genera to markers of brain health, independent of major demographic and vascular risk factors.

**Implications of all the available evidence**

Together with prior work, these findings support the oral microbiome as a relevant and potentially modifiable factor in brain health and cognitive ageing. They highlight periodontitis-related microbial abnormalities as early correlates of subclinical cognitive and brain structural changes, underscoring the importance of oral health for human brain health and motivating longitudinal and interventional studies to assess causality and preventive potential.

## Introduction

The human oral microbiome harbours a diverse community of microorganisms that influences the health and well-being of their hosts.^1^ In recent years, periodontitis, which is the inflammatory disruption in the host–microbial homoeostasis of the periodontal pocket, has gained increasing attention as a key factor impacting brain health. Studies indicate that periodontitis and the linked bacterial communities are associated with the incidence of cognitive decline and Alzheimer's dementia.^2^, ^3^, ^4^, ^5^ Given its estimated prevalence between 10% and 50% in elderly people, this renders periodontitis a relevant public health concern.^6^ As therapeutic interventions can alter the progression of periodontitis, comprehending its impact on the brain is vital for effective prevention and management of cognitive sequelae.

Among bacterial species of the subgingival biofilm, several are known to be associated with periodontitis including *Porphyromonas gingivalis*, *Tannerella forsythia* and *Treponema denticola*.^7^^,^^8^ Mechanistic models have been proposed to explain the connection between these bacterial communities and cognitive decline. The connection is considered to arise from bacterial species promoting systemic inflammation, neurodegenerative processes, and blood–brain barrier disruption.^9^^,^^10^

Despite existing research efforts, our understanding of the association between oral bacterial communities and brain health remains limited. While recent microbiome studies have predominantly focused on clinical Alzheimer's disease and mild cognitive impairment cohorts,^5^^,^^11^^,^^12^ studies trying to understand these connections in the general population remain scarce.^13^ Additionally, many investigations are constrained by small sample sizes and a lack of multi-modal imaging and biomarker data potentially leading to inconsistent findings.^5^ These issues are further complicated by the inherent complexity of microbiome data.

Tapping into these research needs, we aim to advance the understanding of the oral microbiome–brain axis by applying advanced analysis techniques capable of integrating the large-scale, multi-domain data crucial for achieving reliable and actionable insights. Specifically, our primary hypothesis was that subgingival bacterial profiles indicative of periodontitis would be associated with variations in brain health-related host phenotypes within a population-based cohort. To investigate this hypothesis, we analysed population-based data of n = 1026 individuals from the PAROMIND Study.^14^ Our approach focuses on a topology-based analysis that integrates relative abundance data of subgingival microbiota derived from 16S rRNA gene amplicon sequencing of subgingival samples with in-depth clinical and lifestyle data including oral health assessments, cognitive test scores, mental health scores, neuroimaging, circulating inflammatory markers, dietary patterns, and vascular risk measures.

## Methods

### Study design

**Fig. 1:**
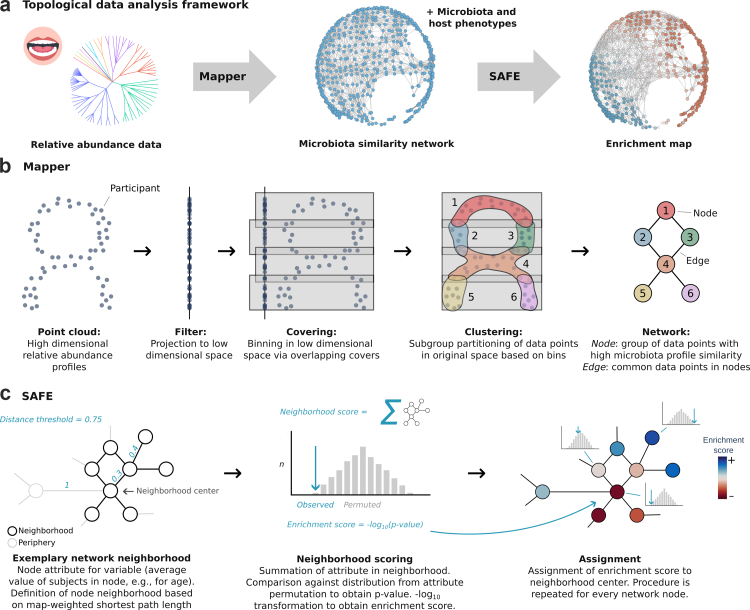
**Methodology.** a) Topological data analysis framework. Genus-level subgingival microbiota relative abundance data were used as input for the Mapper algorithm, which constructed a microbiota similarity network. This network was then annotated with microbiota and host phenotypes using Spatial Analysis of Functional Enrichment (SAFE), yielding enrichment maps that highlight regions of the network where specific attributes are significantly higher or lower than expected by chance. Data shown in this panel is exemplary and based on simulations. b) We applied the Mapper algorithm to transform the high-dimensional genus-level subgingival microbiota profiles into an interpretable low-dimensional network representation. Mapper consists of multiple steps: (1) Inputting participant-level genus abundance; (2) Projecting data points to a low-dimensional space using a filter function for dimensionality reduction; (3) Dividing the low-dimensional space into overlapping covers, each containing a subset of data points; (4) Clustering data points within each cover based on their distances in the original high-dimensional space; (5) Constructing a network from the clustering results, where each node represents a cluster of participants, and links between nodes indicate shared participants between clusters. Modified from Liao et al.^15^ c) Spatial Analysis of Functional Enrichment (SAFE) was used to annotate the network derived via Mapper, identifying regions significantly enriched with specific attributes (e.g., systolic blood pressure). SAFE involves the following steps: (1) Computing node attributes by averaging variables of interest across participants within each node; (2) Defining the local neighbourhood by identifying all nodes within a maximum distance threshold from the centre node, with distance measured by map-weighted shortest path length (MSPL); (3) Calculating a neighbourhood score by summing the attribute values of neighbouring nodes; (4) Computing a p-value by comparing the empirical neighbourhood score against a randomly permuted distribution, achieved through random node-to-attribute reassignments while preserving network topology; (5) Assigning an enrichment score to the neighbourhood centre using -log10 transformation of the multiple testing-corrected p-value. This procedure is repeated for each node, resulting in an enrichment map indicating where attributes are higher or lower than expected by chance. Modified from Baryshnikova et al.^16^*Abbreviations*: BOP index, bleeding on probing index; clin., clinical; cort., cortical; DMFT index, decayed, missing, filled teeth index; hsCRP, high-sensitivity c-reactive protein; subcort., subcortical.

This analysis integrates subgingival microbiota composition with oral health, cognitive, mental health, neuroimaging, inflammatory, dietary, vascular, and demographic phenotypes from the PAROMIND Study. The analytical framework comprised two stages: (1) a primary, exploratory topological data analysis (TDA) using the Mapper algorithm and SAFE enrichment analysis to characterise the microbiome structure and its co-variation with phenotypes (Fig. 1a); and (2) a secondary, covariate-adjusted group comparison to formally test key associations. Full details on the study methodology are provided in the Supplementary Methods.

### Study population

PAROMIND is a cross-sectional study nested within the Hamburg City Health Study (HCHS). The HCHS is a prospective, single-centre, population-based cohort study investigating adults aged 45–75 to enhance the detection of major chronic disease risks through extensive clinical and imaging phenotyping.^14^ Participants were randomly selected from the official resident registry using stratified sampling by age and sex and contacted via an invitation letter sent to their home address. Participants were included in PAROMIND if they had no requirement for endocarditis prophylaxis, and a full dental examination has been performed. The study protocol includes assessments of oral health including collection of gingival crevicular fluids for microbiome analyses, cognition, mental health, diet, vascular risk, brain MRI, and blood sampling. For each participant, the clinical assessments and biological sampling were generally completed during a single comprehensive baseline visit; the separate brain MRI appointment followed shortly thereafter, ensuring reasonable contemporaneity between these measures for cross-sectional analysis. Participants were excluded due to poor data quality, missing microbiome data, or unavailable imaging data.

### Ethics statement

PAROMIND and the HCHS were approved by the local ethics committee of the Landesärztekammer Hamburg (State of Hamburg Chamber of Medical Practitioners, PV5131). The conduct of PAROMIND is governed by ethical guidelines of Good Clinical Practice (GCP), Good Epidemiological Practice (GEP) and the Declaration of Helsinki. Written informed consent was obtained from all participants prior to study enrolment.

### Phenotyping

#### Oral health assessment

Oral health was assessed by a certified study nurse following a standardised protocol.^17^ Study nurses were trained and certified by licenced dentists experienced in periodontal examination procedures prior to data collection. Clinical attachment loss (CAL) was measured at six sites per tooth (mesio-buccal, buccal, disto-buccal, disto-palatinal, palatinal and mesio-palatinal) using a standard periodontal probe.^18^ Additionally bleeding on probing (BOP) index and DMFT index were recorded. The plaque index (PI) was measured at two interproximal sites per tooth to evaluate dental plaque accumulation. The periodontal inflamed surface area (PISA) was adapted to the HCHS protocol.^19^, ^20^, ^21^ The oral health assessment followed a standardised protocol for the reporting of the prevalence and severity of periodontal diseases.^22^ Periodontitis severity was classified based on the “Application of the 2018 periodontal status Classification to Epidemiological Survey data” (ACES) framework.^23^^,^^24^

#### Oral microbiota

Subgingival microbiota profiling targeted gingival crevicular fluid collected from the four deepest periodontal pockets across the dentition (pooled paper-point sample, 15 s in situ per paper point, sterile 2 ml Eppendorf tube, no transport medium), stored at −80 °C until further processing. Deepest pockets were identified based on the recorded probing depth. No minimum probing depth was required. This ensured that every participant contributed a four-point pooled subgingival sample.

Samples were sequenced at the Institute of Clinical and Molecular Biology (Kiel, Germany) by 16S rRNA gene amplicon sequencing of the V3–V4 region (Illumina MiSeq, 2 × 300 bp; full extraction and sequencing protocol in *supplementary methods*). Data were processed using the DADA2 workflow (v. 1.10, https://benjjneb.github.io/dada2/bigdata.html), with taxonomic annotation against the expanded Human Oral Microbiome Project database (eHOMD v15.23). Samples with fewer than 10,000 sequences were excluded. Analyses were conducted at genus level, discarding genera present at <0.1% relative abundance, balancing statistical power, interpretability, and dimensionality.

#### Cognitive and mental health assessments

Cognitive function was assessed using the extended CERAD-NP/Plus battery,^25^ administered by a trained study nurse. Individual test scores (Trail Making Tests A and B, Word List Recall, Multiple Choice Vocabulary Intelligence Test B, Animal Naming Test, Mini Mental State Exam) were combined into a general cognitive ability index (*g*) via principal component analysis (first component, 40.5% variance explained; details including scree and biplot in Supplementary Figure S1),^26^ with scores inverted so that higher values reflect better performance. Mental health was assessed via validated questionnaires: PHQ-9, Geriatric Depression Scale (depression), PHQ-15 (somatic symptoms), and GAD-7 (anxiety).^27^, ^28^, ^29^

#### Neuroimaging of brain macro- and microstructure

Neuroimaging markers of macro- and microstructural brain integrity were derived from T1-weighted and diffusion-weighted MRI following established procedures (acquisition, preprocessing, and quality assessment are detailed in the Supplementary Methods).^30^ Cortical thickness and subcortical volume were estimated using FreeSurfer (v6.0.1) and combined into a single z-scored summary measure. Free-water imaging yielded global grey and white matter free-water and tissue fractional anisotropy (FA), reflecting extracellular water accumulation and neurite integrity, respectively.^31^

#### Diet

Dietary behaviour was assessed using a validated 102-item food frequency questionnaire developed for the EPIC Study.^32^ Adherence to the Mediterranean (MEDAS), DASH, and MIND dietary patterns was scored using established methods,^33^, ^34^, ^35^ yielding total adherence scores for each pattern.

### Topological data analysis framework

We implemented a two-component TDA pipeline in Python (v3.8.1; *tmap* v1.2, *safepy*, *NetworkX* v2.2, *scikit-learn* v1.5.1) and R (v4.4.0; *vegan* v2.6–6.1): (1) the Mapper algorithm to reconstruct a microbiota similarity network from genus-level relative abundance data, and (2) SAFE to test phenotype enrichment on this network.^15^^,^^16^

#### Mapper

The applied Mapper pipeline comprises multiple analysis steps to reconstruct the topological network (Fig. 1b): filtering, covering, clustering and network reconstruction.^36^ First, as the filtering step, we conducted a principal coordinate analysis of the Aitchison distance of genus-level relative abundance (n_participants_ x n_genera_).^37^ To account for the compositional nature of the data and handle zero values, the distance was calculated using the robust Aitchison method.^38^ By that, we obtained two components—also called lenses—capturing the major axes of variation in the microbial community composition across the participants. Based on these axes, overlapping covers were defined (overlap = 1.5, resolution = 30) to segment the data into overlapping bins, each representing a local region of inter-individual variation. Unsupervised clustering of data points within each bin was performed using Hierarchical Density-Based Spatial Clustering of Applications with Noise (HDBSCAN, epsilon threshold = 0.95).^39^ By this, nodes are obtained that represent participant groups with similar configuration of the subgingival microbiota. Participants can belong to multiple nodes with the number varying per participant. Lastly, the network reconstruction was accomplished by connecting clusters sharing common participants. Not all data points are retained by Mapper resulting in the omission of some participants (n_not retained_ = 109).

#### SAFE: enrichment analysis

After the network structure was determined by the Mapper algorithm, SAFE was applied to evaluate phenotype enrichment on this fixed topology. SAFE is an annotation technique for biological networks that enables the computation and statistical assessment of local enrichment for specific phenotypes.^16^ In our work, we performed SAFE to identify regions within the microbiota similarity network that are significantly enriched for specific participant traits. Specifically, we investigated the enrichment of genus-level relative abundance, oral health measures (clinical attachment loss, plaque index, bleeding on probing index, DMFT index, PISA, missing teeth, ACES classification), cognitive scores (general cognitive ability, Animal Naming Test, Mini Mental State Exam, Multiple Choice Vocabulary Intelligence Test B, Trail Making Tests A and B, Word List Recall), mental health scores (PHQ-9, PHQ-15, Geriatric Depression Scale, GAD-7), imaging measures (cortical thickness and subcortical volume, grey matter free-water, white matter free-water, grey matter tissue fractional anisotropy, white matter tissue fractional anisotropy), circulating inflammatory markers (hsCRP, leukocytes), diet scores (MEDAS score, DASH score, MIND score), vascular risk factors (systolic and diastolic blood pressure, body mass index, smoking behaviour, blood triglycerides, cholesterol, low density lipoprotein, high density lipoprotein, HbA1c), and demographics (age, sex, education).

Following previous analyses leveraging SAFE,^16^^,^^40^ the microbiota similarity network was spring-embedded, i.e., nodes in the network were positioned so that those with connections are placed closer together, while repulsive forces push non-connected nodes apart, resulting in a visually balanced and interpretable representation of the network's structure.^41^ Next, we computed node attributes for all phenotypes by averaging the values of each variable across all participants within a node.

Enrichment scores were computed for each node by comparing observed neighbourhood attribute values against a null distribution from 5000 permutations (Fig. 1c; details in Supplementary Methods and^16^) (MSPL distance threshold = 0.75). Significance was defined as FDR-corrected p < 0.05, corresponding to an enrichment score threshold of −log_10_ (0.05) = 1.30. Note that 5000 permutations impose a practical upper bound on the enrichment score of −log_10_ (1/5000) = 3.70, as this is the smallest p-value achievable given the permutation resolution. Positive enrichment scores indicate that observed values are higher than the permuted distribution, negative enrichment scores indicate that they are lower than the permuted distribution. This procedure is repeated for each node of the network, resulting in an enrichment map indicating where attributes are higher or lower than expected by chance.

To understand the primary taxonomic patterns shaping the network topology, we performed a dominance analysis alongside examining individual phenotype enrichments. This involved labelling each network node by the bacterial genus with the highest positive enrichment score. Visualising this node-level dominance helps to reveal major taxonomic transitions across the network. While highlighting the most strongly enriched genus in each region provides valuable pointers to key drivers, this is a simplification; each node still represents a complex microbial community, not just the dominant genus identified.

To quantify how strongly the network topology captures each phenotype, we computed the enrichment ratio (proportion of significantly enriched nodes, FDR-corrected p < 0.05). To corroborate enrichment-based analyses using complementary, distance-based approaches and to quantify the proportion of microbiome variance explained by each phenotype, we tested phenotype–microbiome associations using envfit and PERMANOVA (both: 5000 permutations; FDR-corrected) on the robust Aitchison distance matrix via *vegan*. To identify phenotypes that independently explained microbiome variance, we performed forward model selection via distance-based redundancy analysis (db-RDA; *ordiR2step*, 5000 permutations), with missing values imputed by KNN (k = 5) prior to fitting. Co-enrichment structure across phenotypes was assessed by PCA of enrichment scores and pairwise Spearman correlation.

To determine whether specific phenotypes co-enrich, i.e., exhibit similar enrichment patterns, we performed an ordination of the enrichment scores using principal component analysis retaining the first two principal components (explained variance: 73.17%) and assessed the pairwise Spearman correlation between the enrichment scores.

### Group analysis

To formally test whether the associations between microbiome-derived group membership and brain health phenotypes are independent of potential confounders we performed a secondary group analysis with covariate adjustment. Group characteristics are detailed in Supplementary Table S1.

Nodes of the microbiota similarity network were assigned to two groups using k-Means clustering (k = 2; justified by the linear gradient evident in enrichment analysis). Participants assigned to nodes in both groups (n = 137, representing the intermediate transition zone) were excluded to ensure statistical independence. Participants not retained by Mapper (n = 109) were also excluded, as their microbiome profiles did not form sufficient clusters within the Mapper reconstruction and were therefore not assigned to any node. Comparisons of demographic, clinical, cognitive, inflammatory, and brain structural variables between retained and excluded participants at both stages confirmed that the exclusions did not introduce systematic bias with respect to the primary outcome variables (Supplementary Tables S2 and S3).

Groups were compared using multiple linear regression, with age, sex, education, and vascular risk factors (systolic/diastolic blood pressure, BMI, smoking, triglycerides, total cholesterol, LDL, HDL, HbA1c) included as pre-specified covariates^17^^,^^42^, ^43^, ^44^:

Phenotype∼Group+Age+Sex+Education+Vascularriskfactors

An additional sequential covariate model was run to quantify individual confounder contributions. Resulting regression coefficients are reported as standardised betas (β_std_) which express the between-group difference in standard deviation units of the dependent variable. Robustness of group comparison results was assessed using case-resampling bootstrap regression (10,000 iterations) with bias-corrected and accelerated confidence intervals.

### Sensitivity analysis

To verify robustness to pipeline parameter choices, we systematically varied Mapper cover overlap (1.0–2.0), resolution (20–45), and epsilon threshold (0.90–0.99), and SAFE distance threshold (0.5–0.99) and neighbourhood radius (0.05–0.15), one parameter at a time (17 iterations total). Robustness was evaluated by Spearman correlation of enrichment ratios and Adjusted Rand Index (ARI) of group assignments across configurations.

### Role of the funding source

The funders had no role in the design of the study, the collection, analysis, or interpretation of the data, or in the preparation of this manuscript.

## Results

Quality assessment of microbiome and brain MR imaging data resulted in a final analysis sample of 1026 individuals ([mean ± SD] age 63.72 ± 8.2 years, 42.7% female; for details see Table 1). A flow chart documenting the sample selection process is shown in Supplementary Figure S2. Sample sizes for individual clinical and lifestyle phenotypes vary across the cohort due to naturally occurring missing data (e.g., participants declining specific assessments, incomplete questionnaires, or low-quality imaging data).

**Table 1:** Sample characteristics.

Metric	Value^a^	Range
Age, years	63.72 ± 8.17 (1026)	46–78^b^
Female, n (% female)	438 (42.7)	
Education, ISCED	2.42 ± 0.58 (999)	1–3
Oral health measures		
Clinical attachment loss	2.61 ± 0.88 (1025)	1.22–8.86
Plaque index	7.69 [0, 29.0] (1014)	0–100
Bleeding on probing index	8.7 [2.1, 19.6] (1011)	0–100
DMFT index	19.27 ± 5.02 (1026)	5–28
PISA	90.98 [21.0, 230.2] (1010)	0–2672
Missing teeth	3 [1, 6] (1025)	0–28
ACES Periodontitis Classification, n (%)	Periodontal health: 2 (0.2) Localised gingivitis: 0 (0) Generalised gingivitis: 0 (0) Stage I: 9 (0.9) Stage II: 315 (30.7) Stage III: 467 (45.5) Stage IV: 231 (22.5) Edentulism: 0 (0) Non-classified: 0 (0)	
Cognitive scores		
Animal Naming Test	24.66 ± 6.67 (977)	0–50
Mini Mental State Exam	27.85 ± 1.68 (973)	19–30
Multiple Choice Vocabulary Intelligence Test	31.42 ± 3.43 (838)	9–37
Trail Making Test A, sec	39.82 ± 14.07 (925)	13–104
Trail Making Test B, sec	89.44 ± 37.66 (917)	29–281
Word List Recall	7.70 ± 1.88 (949)	0–10
Mental health scores		
PHQ-9	2 [0, 5] (1025)	0–22
PHQ-15	4.27 ± 3.90 (1025)	0–21
Geriatric Depression Scale	1 [0, 3] (914)	0–14
GAD-7	1 [0, 4] (1025)	0–20
Global neuroimaging features		
Free-water (grey matter)	0.50 ± 0.05 (909)	0.38–0.67
Free-water (white matter)	0.31 ± 0.03 (909)	0.24–0.41
Tissue fractional anisotropy (grey matter)	0.19 ± 0.01 (909)	0.16–0.21
Tissue fractional anisotropy (white matter)	0.41 ± 0.01 (909)	0.38–0.44
Mean cortical thickness, mm	2.63 ± 0.11 (986)	1.62–2.94
Mean subcortical volume, ml	3188.31 ± 313.66 (986)	1725.70–4091.91
Inflammatory markers		
hsCRP	0.11 [0.06, 0.25] (970)	0.01–5.76
Leukocytes	6.13 ± 1.75 (1006)	2.3–21.4
Diet		
MEDAS score	4.46 ± 1.89 (970)	1.5–8.5
DASH score	4.46 ± 1.06 (970)	0–11
MIND score	6.30 ± 1.78 (970)	1–11.5
Vascular risk measurements		
Systolic blood pressure	142.12 ± 20.06 (997)	87.5–226.5
Diastolic blood pressure	83.46 ± 10.55 (997)	50.5–128.0
Body mass index	26.60 ± 4.32 (995)	17.63–47.19
Smoking, %	173 (16.9)	
Triglycerides, mg/dl	117.67 ± 75.68 (1007)	31.0–1054.0
Cholesterol, mg/dl	208.28 ± 40.83 (1008)	100.0–366.0
LDL, mg/dl	121.56 ± 36.53 (997)	24.0–269.0
HDL, mg/dl	63.51 ± 19.00 (1008)	18.0–153.0
HbA1c	5.63 ± 0.54 (1007)	4.5–10.0

Abbreviations: DMFT index, decayed/missing/filled teeth index; HDL, high density lipoprotein; hsCRP, high sensitivity c-reactive protein; IQR, interquartile range; LDL, low density lipoprotein; ISCED, International Standard Classification of Education; mm, millimeters; sec, seconds.

a Continuous data are presented as mean ± standard deviation (SD) or, for skewed distributions, as median and interquartile range [IQR]. Categorical data are presented as absolute numbers and percentages (%). The number of available observations (N) is indicated for each variable.

b Note: While the target age for study recruitment is 45–75 years, the actual age of some participants could differ due to the time elapsed between the initial invitation and the examination appointment.

### A similarity network of subgingival bacterial communities

**Fig. 2:**
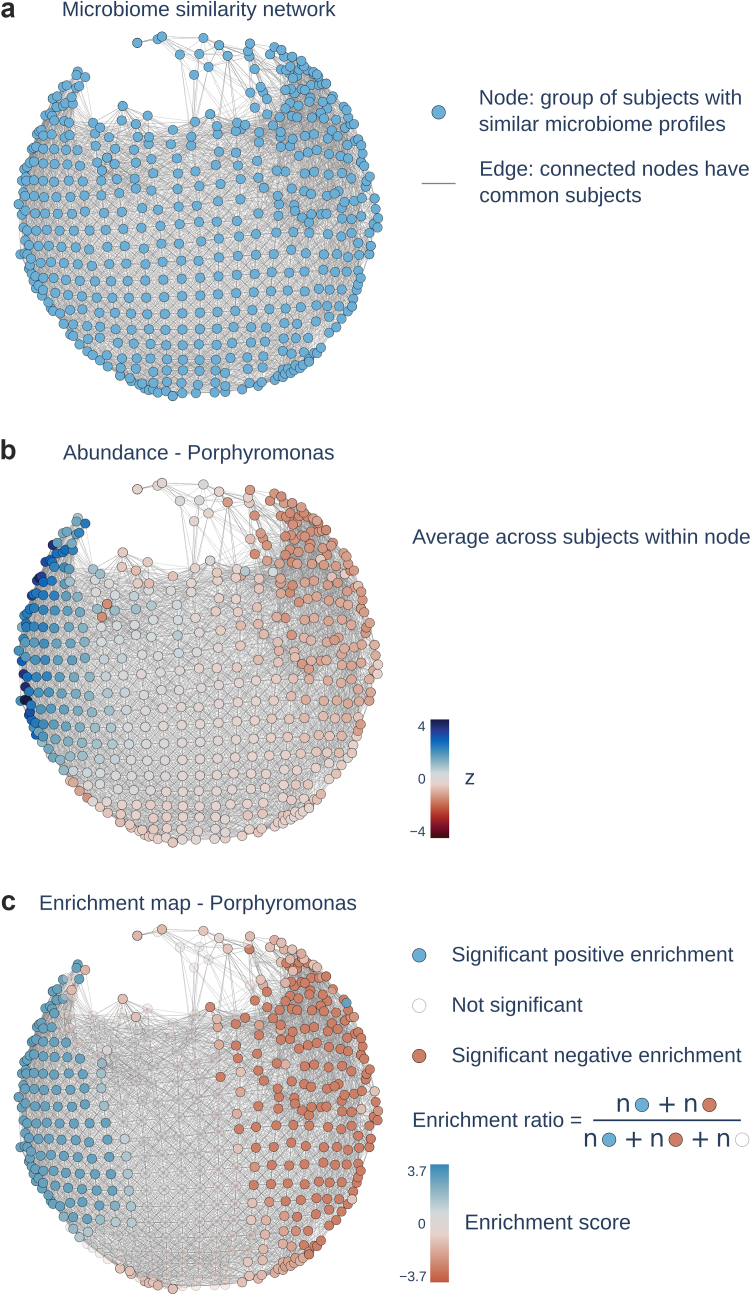
**Microbiota similarity network based on genus-level relative abundance.** a) Microbiota similarity network obtained by applying the Mapper algorithm to the relative abundances of bacterial genera consisting of 577 nodes and 10230 edges. Nodes represent groups of participants with similar microbiota profiles. Edges connect nodes that share common participants. Within the network, proximity signifies microbiome profile similarity among participants. b) Exemplary network distribution of z-scored *Porphyromonas* abundance. *Porphyromonas* was selected given its high relevance in periodontal disease.^45^ c) Enrichment map of *Porphyromonas* abundance. Nodes with positive enrichment scores are coloured blue indicating a higher *Porphyromonas* abundance of the node and its neighbourhood than expected by random permutation. Nodes with negative enrichment scores are coloured red indicating lower *Porphyromonas* abundance than expected by permutation. Non-significant nodes are shown in a lighter shade. The enrichment ratio represents a measure of how strongly a phenotype is enriched on a network. It is computed by dividing the count of significantly enriched nodes by all nodes.

We applied an unsupervised, topology-based technique to relative abundance data of 85 different bacterial genera to infer a low-dimensional network representation of oral microbiota similarity. The resulting microbiota similarity network consisted of 577 nodes—representing participant groups with highly similar subgingival microbiota compositions—and 10,230 edges—connecting nodes that share at least one participant. The network represents a map of inter-individual variability capturing transitions in microbial composition across the cohort. For a visualisation of the network see Fig. 2a.

In an enrichment analysis, we examined whether the microbiota similarity network captures inter-individual differences in participant traits. Therefore, we computed enrichment scores, which are node-level indices that indicate whether participant traits are significantly higher or lower than expected by chance in specific regions, i.e., participant clusters, of the network. Enrichment scores were obtained for genus-level relative abundance data as well as all non-microbiome phenotypes. Furthermore, we calculated the enrichment ratio (= number of significantly enriched nodes/total number of nodes) based on the enrichment scores for each phenotype quantifying the overall amount of enrichment on the microbiota similarity network. This measure indicates how strongly the network-based organisation of participants reflects inter-individual variance in a respective phenotype. Refer to Fig. 2b and c for an exemplary display of *Porphyromonas* abundance and the corresponding enrichment map on the microbiota similarity network.

### Enrichment analysis of microbiome phenotypes

**Fig. 3:**
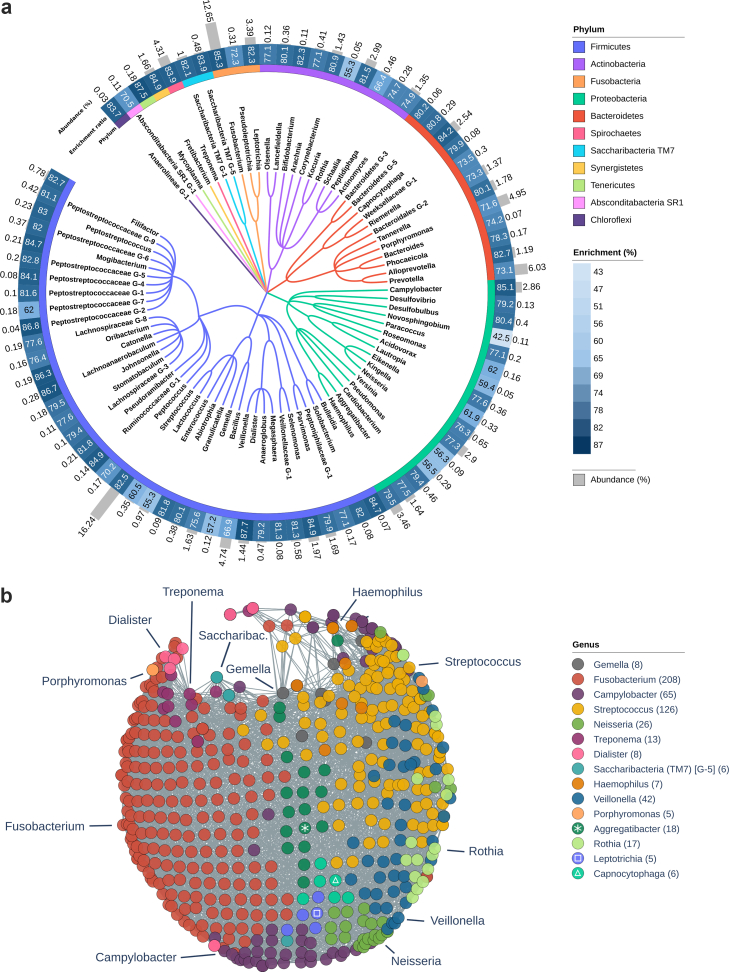
**Enrichment analysis of microbiome phenotypes.** a) Circular phylogenetic tree at the level of oral microbiome genera. The inner band shows phyla with corresponding colouring of the tree, the mid band displays the enrichment ratio and the outer band shows overall relative abundance. The phylogenetic tree was visualised using iTOL (v6). b) Dominance analysis: Nodes of the microbiota similarity network are coloured to represent the bacterial genus with the highest enrichment score. Genera with dominance in five or more nodes are shown. *Abbreviations*: GM, gray matter; WM, white matter.

Genus-level relative abundances showed significant enrichment ranging from 42.5% to 87.7%. Fig. 3a displays the enrichment ratios alongside mean relative abundance and phylogenetic associations. We performed a dominance analysis by identifying for each network node the bacterial genus with the highest enrichment scores (Fig. 3b). Of the 85 investigated genera, 15 showed the highest enrichment score for at least 5 nodes. Distribution of dominant genera in the network representation of oral microbiota similarity followed a horizontal pathogenicity gradient: Bacterial genera with strongest enrichments at the *left* end of the microbiota similarity network were periodontitis-associated taxa including *Fusobacterium* (number of nodes with highest enrichment score—n_nodes_ = 208), *Campylobacter* (n_nodes_ = 65), *Treponema* (n_nodes_ = 13), *Dialister* (n_nodes_ = 8), *Saccharibacteria* (TM7) [G-5] (n_nodes_ = 6) and *Porphyromonas* (n_nodes_ = 5). In the centre of the network, *Aggregatibacter* (n_nodes_ = 18), *Gemella* (n_nodes_ = 8), *Capnocytophaga* (n_nodes_ = 6) and *Leptotrichia* (n_nodes_ = 5) exhibited the highest enrichment scores. At the right end, genera with strongest enrichments were of low periodontal pathogenicity or related to other dental diseases including *Streptococcus* (n_nodes_ = 126), *Veillonella* (n_nodes_ = 42), *Neisseria* (n_nodes_ = 26), *Rothia* (n_nodes_ = 17) and *Haemophilus* (n_nodes_ = 7).

### Enrichment analysis of host phenotypes

**Fig. 4:**
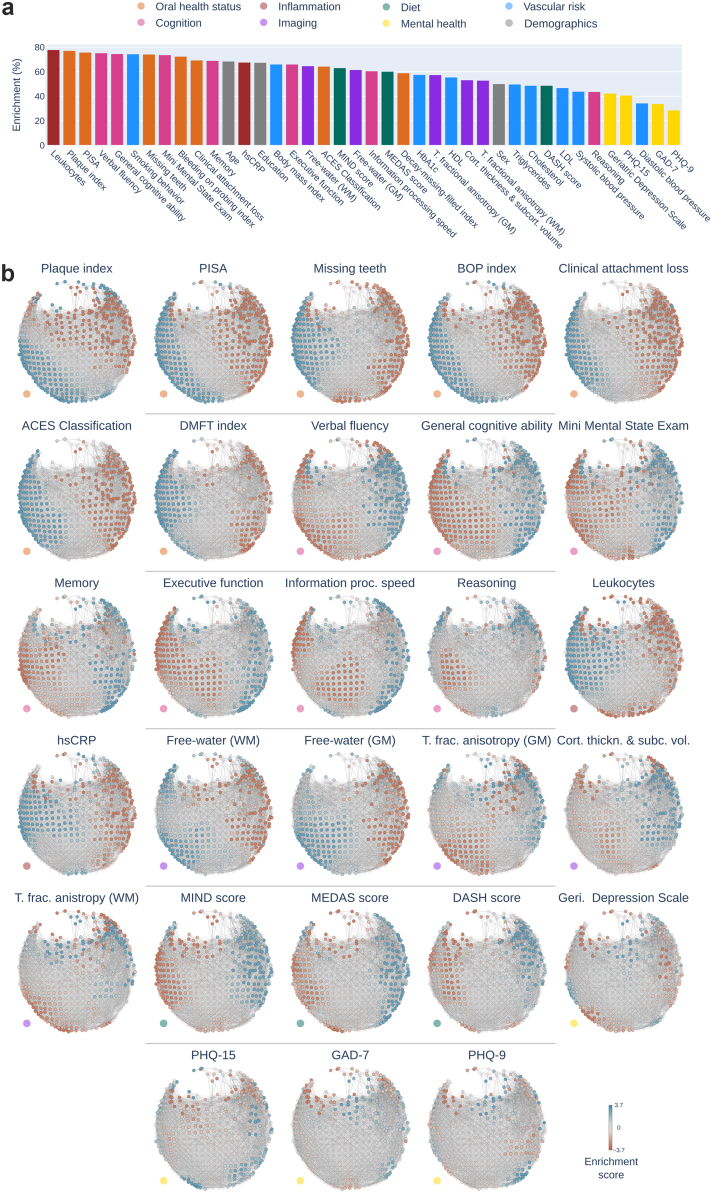
**Enrichment analysis of non-microbiome phenotypes.** a) The bar plot displays the enrichment ratio (= significantly enriched network nodes/all network nodes) of non-microbiome phenotypes. The enrichment ratio reflects how strongly a phenotype's variance is captured by the topology of the microbiome network obtained via Mapper. The bars of the plot are coloured by variable category. b) Enrichment maps: Enrichment scores (-log_10_-transformed p-values) are mapped on the microbiota similarity network. These maps visualise how specific clinical and lifestyle traits are distributed across the different microbial community profiles. Nodes with positive enrichment scores, indicating a significantly higher phenotype value in that network region than expected by chance, are coloured blue. Conversely, nodes with negative enrichment scores, indicating a significantly lower phenotype value than expected by chance, are coloured red. Non-significant nodes are shown in a lighter shade. The spatial distribution of blue and red regions indicates whether a specific trait (e.g., elevated leukocytes) is concentrated in a particular ‘microbial neighbourhood’ or follows a continuous gradient across the population. Non-microbiome phenotypes are first ordered by domain (denoted by coloured dots in the lower-left) and then by enrichment ratio. Enrichment maps for demographics, vascular risk factors and genus-level relative abundances are not displayed and can be found in the online supplement (https://osf.io/vqj8m/). *Abbreviations*: BOP index, bleeding on probing index; DMFT index, decayed, missing, filled teeth index; GM, gray matter; hsCRP, high-sensitivity c-reactive protein; LDL, low density lipoprotein; T. fractional anisotropy, tissue fractional anisotropy; WM, white matter.

All non-microbiome phenotypes showed significant enrichment on the microbiota similarity network including measures of oral health status, cognition, mental health, brain structure, circulating inflammatory markers, diet, vascular risk factors and demographics (Fig. 4). The top 10 ranking non-microbiota phenotypes were leukocytes (78.0%), plaque index (77.3%), periodontal inflamed surface area (75.9%), verbal fluency (75.4%), general cognitive ability (74.7%), smoking behaviour (74.5%), missing teeth (74.3%), Mini Mental State Exam (73.8%), bleeding on probing index (72.6%), and clinical attachment loss (69.5%) (Fig. 4a). A complementary analysis using envfit and adonis confirmed these results: Among the phenotypes with the top 10 highest enrichment ratios, all showed a significant association (p_FDR_ < 0.05) with subgingival microbiota profiles (Supplementary Figure S3). Moreover, the enrichment ratio was significantly correlated with the adjusted R^2^ resulting from envfit (Spearman ρ = 0.79, p < 0.001) and adonis (Spearman ρ = 0.83, p < 0.001).

To further disentangle the contributions of non-microbiome variables, we performed a forward model selection (ordiR2step) to identify the set of phenotypes that best explain the variance in microbiota composition (Supplementary Figure S4). Among significantly associated phenotypes (p < 0.05), clinical attachment loss was the strongest contributing factor, explaining 1.72% of the variance, followed by smoking behaviour (+0.58%) and periodontal inflamed surface (+0.52%), ACES classification (+0.49%), missing teeth (+0.29%), plaque index (+0.22%), leukocytes (+0.15%), cortical thickness and subcortical volume (+0.08%), sex (+0.07%), Mini Mental State Exam (+0.07%), and PHQ-15 (+0.04%). Adding the remaining non-microbiome phenotypes explained an additional 0.05%. Collectively, the final model explained approximately 4.29% of the total variance in the microbiome data.

Based on the enrichment maps, we assessed inter-participant differences of phenotypes. Phenotype enrichment maps are presented in Fig. 4b. Most participant traits varied along the microbiota similarity network in a linear left-right trajectory aligning with the pathogenicity gradient identified via dominance analysis.

Put differently, there was a gradient of enrichment from left to right that reflected the transition of participant characteristics. At the individual level, a participant's position on this map reflects their specific clinical profile: participants at the left end of this gradient exhibited higher severity of clinical periodontitis, elevated circulating inflammatory markers, older age, a higher percentage of smokers (indicated by significant positive enrichment), as well as lower cognitive function, lower brain structural integrity, a less healthy diet, a lower percentage of females, and lower education levels (indicated by significant negative enrichment). Conversely, the right end of the gradient featured participants with opposite traits: lower severity of clinical periodontitis, lower circulating inflammatory markers, younger age, fewer smokers, as well as higher cognitive performance, higher brain structural integrity, a healthier diet, a higher percentage of females, and higher education levels. Mental health scores and vascular risk factors beyond smoking displayed a non-linear enrichment pattern not aligned with the pathogenicity gradient.

### Microbiome and host phenotypes co-enrich

**Fig. 5:**
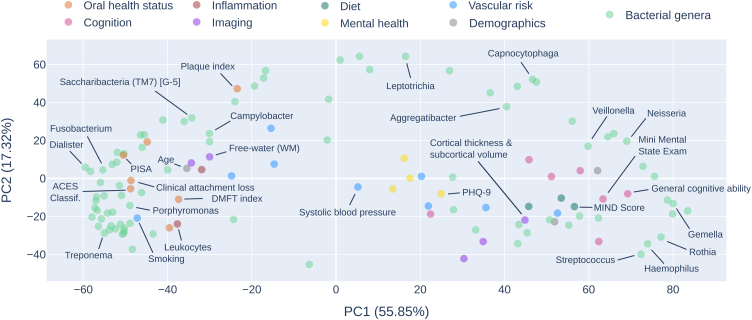
**Principal component analysis of enrichment scores.** The plot illustrates the phenotypes' location in principal component space, where proximity suggests similarity in enrichment patterns. Points are colour-coded by phenotype category. Dominant genera and selected non-microbiome measures from each phenotype group were highlighted with annotations. For a fully annotated scatter plot, refer to the supplementary materials.

**Fig. 6:**
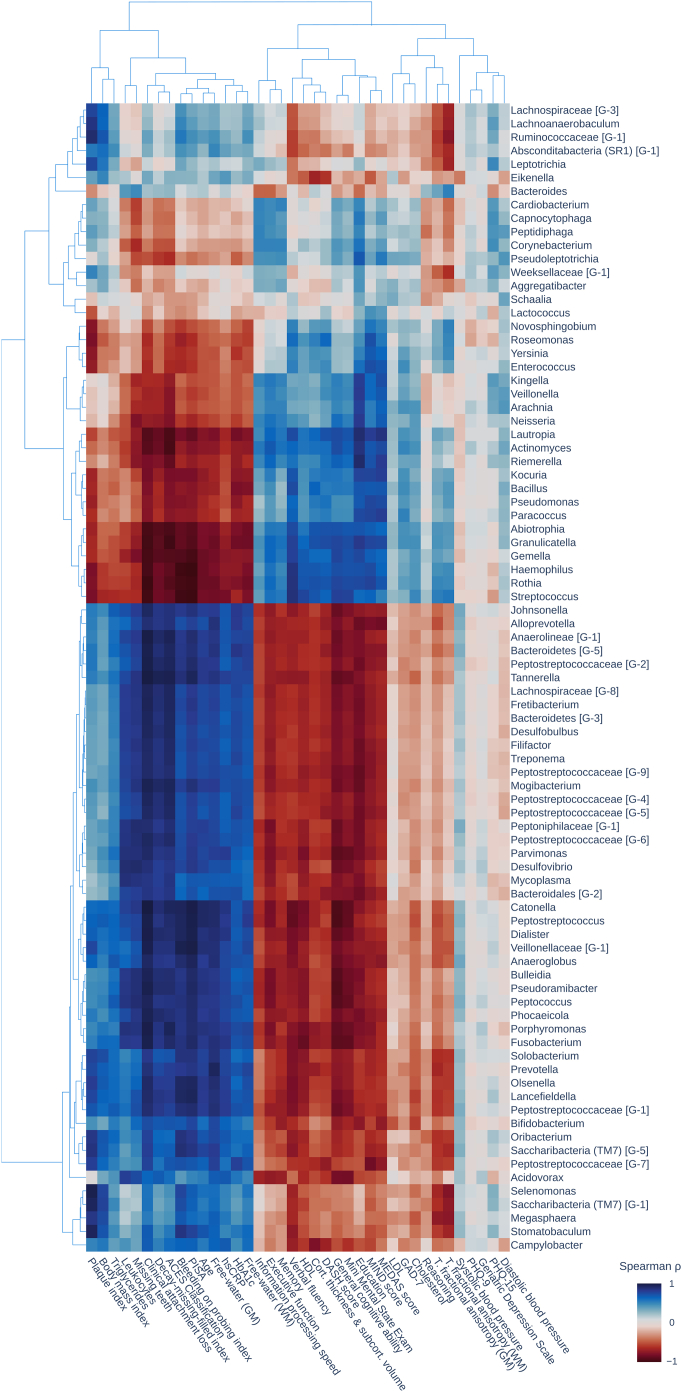
**Co-enrichment heatmap.** The heatmap presents the Spearman correlation of enrichment scores for genus-level relative abundance and non-microbiome phenotypes. The order is determined by hierarchical clustering of the enrichment scores. *Abbreviations*: BOP index, bleeding on probing index; DMFT index, decayed, missing, filled teeth index; GM, gray matter; LDL, low density lipoprotein; T. fractional anisotropy, tissue fractional anisotropy; WM, white matter.

Principal component analysis of enrichment scores revealed that enrichment patterns of phenotypes differed along two dominant axes of inter-phenotype variation explaining 55.85% (principal component 1, PC1) and 17.32% (principal component 2, PC2) of variance, respectively. Phenotypes with similar enrichment patterns co-localised in the principal component space formed by these axes (Fig. 5, Supplementary Figure S5). Phenotypes with enrichment patterns indicating increasing values from right end of the network to left were localised on the left extreme of the principal component space including periodontitis-associated bacterial genera, clinical oral health measurements, circulating inflammatory markers, smoking behaviour and white matter free-water measured by diffusion-weighted MRI. Phenotypes increasing left to right were located on the right including health-associated bacterial genera, cognitive performance measurements, diet scores, cortical thickness and subcortical volume as measured by MRI. Phenotypes showing a non-linear enrichment pattern or transition pattern in the left-right right-left orientation were localised in the middle including mental health scores and vascular risk measurements apart from smoking. For a heatmap depicting the Spearman correlation of enrichment scores for genus-level relative abundance and non-microbiome phenotypes see Fig. 6. For the same co-enrichment heatmap indicating genus–genus correlation as well as correlation between non-microbiome phenotypes see Supplementary Figures S6 and S7.

### Microbiota similarity groups differ in periodontal disease, inflammatory markers, cognition and diet

**Fig. 7:**
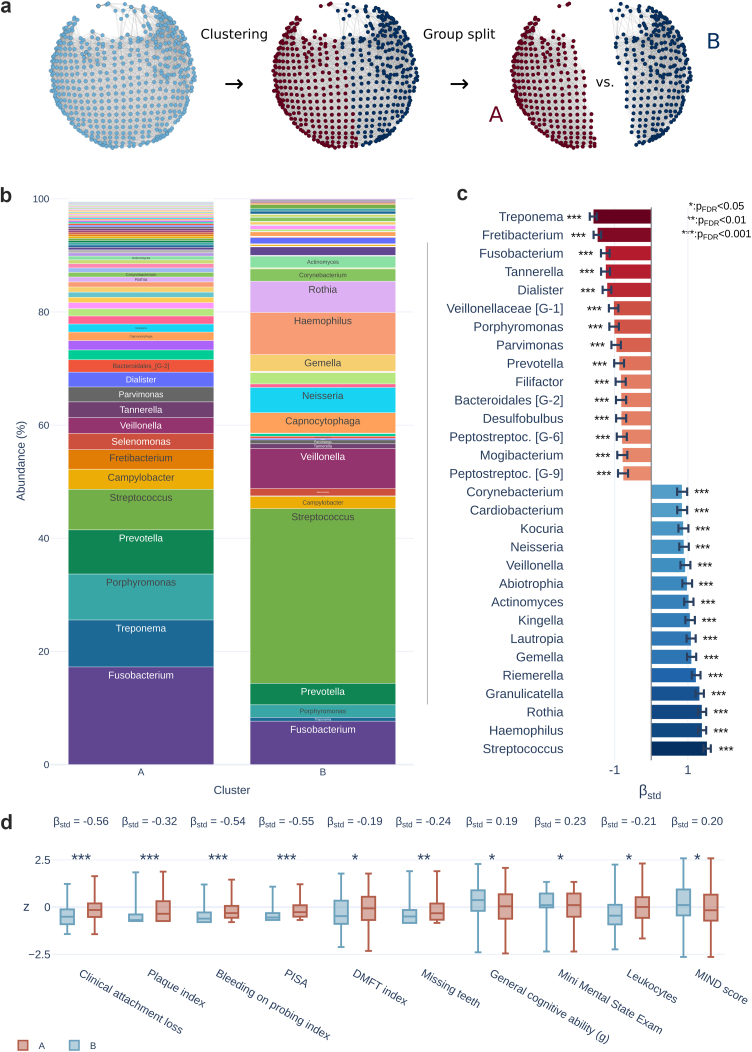
**Group analysis.** a) Approach: The nodes of the microbiota similarity network were divided into two distinct groups (“A” and “B”) using k-means clustering. Participants were categorised based on these groupings, with individuals present in both groups being excluded from the analysis (n = 137). The two participant groups were then statistically compared using linear regression analysis, adjusting for age, sex, education and vascular risk factors. b) Relative abundance of bacterial genera within groups. c) Top 15 positive and negative differences in bacterial genera: Negative regression coefficients (higher in the “A” group) are shown in red, while positive coefficients (higher in the “B” group) are shown in blue. Significance is indicated by asterisks. For a bar plot displaying coefficients of all investigated genera see supplementary materials. d) Shown are box plots for significant associations. Box plot colours correspond to the groups. Statistical significance level is indicated by asterisks. For box plots of the remaining non-microbiome phenotypes see *supplementary materials*. *Abbreviations*: β_std_, standardised beta coefficient for the group variable; BOP index, bleeding on probing index; DMFT index, decayed, missing, filled teeth index; GM, gray matter; *Peptostreptoc.*, *Peptostreptococcaceae*; WM, white matter.

Applying k-means clustering on the microbiota similarity network topology resulted in an unsupervised separation of the sample into two participant groups along the pathogenicity gradient. The following statistics to characterise the identified participant groups were adjusted for potential confounders including age, sex, education and vascular risk factors. The procedure is illustrated in Fig. 7a. Participants assigned to nodes in both groups were excluded from the analysis (n_excluded_ = 137).

We compared genus-level relative abundance to identify bacterial genera that showed significant differences between groups (Fig. 7b and c). Of the 85 tested genera, 10 showed no significant group differences (Supplementary Figure S8). Top 15 bacterial genera which were significantly higher in the “A” group were *Treponema* (β_std_ = −1.58), *Fretibacterium* (β_std_ = −1.47), *Fusobacterium* (β_std_ = −1.25), *Tannerella* (β_std_ = −1.25), *Dialister* (β_std_ = −1.20), *Veillonellaceae* [G-1] (β_std_ = −1.03), *Porphyromonas* (β_std_ = −1.01), *Parvimonas* (β_std_ = −0.96), *Prevotella* [G-2] (β_std_ = −0.87), *Filifactor* (β_std_ = −0.83), *Bacteroidales* [G-2] (β_std_ = −0.82), *Desulfobulbus* (β_std_ = −0.82), *Peptostreptococcaceae* [G-6] (β_std_ = −0.80), *Mogibacterium* (β_std_ = −0.79), and *Peptostreptococcaceae* [G-9] (β_std_ = −0.77) (all p_FDR_ < 0.001). Top 15 bacterial genera which were significantly higher in the “B” group were *Streptococcus* (β_std_ = 1.52), *Haemophilus* (β_std_ = 1.39), *Rothia* (β_std_ = 1.38), *Granulicatella* (β_std_ = 1.32), *Riemerella* (β_std_ = 1.23), *Gemella* (β_std_ = 1.10), *Lautropia* (β_std_ = 1.09), *Kingella* (β_std_ = 1.06), *Actinomyces* (β_std_ = 1.03), *Abiotrophia* (β_std_ = 0.98), *Veillonella* (β_std_ = 0.93), *Neisseria* (β_std_ = 0.90), *Kocuria* (β_std_ = 0.88), *Cardiobacterium* (β_std_ = 0.84), and *Corynebacterium* (β_std_ = 0.84) (all p_FDR_ < 0.001).

In addition, the “A“ group showed significantly higher clinical severity of periodontitis, higher circulating inflammatory markers, lower cognitive performance and lower brain structural integrity compared to the “B” group (Fig. 7d): a significantly higher clinical attachment loss (β_std_ = −0.56, p_FDR_ < 0.001), plaque index (β_std_ = −0.32, p_FDR_ < 0.001), bleeding on probing index (β_std_ = −0.54, p_FDR_ < 0.001), periodontal inflamed surface area (β_std_ = −0.55, p_FDR_ < 0.001), DMFT index (β_std_ = −0.19, p_FDR_ = 0.041), missing teeth (β_std_ = −0.24, p_FDR_ = 0.005), leukocytes (β_std_ = −0.21, p_FDR_ = 0.010) as well as a significantly lower general cognitive ability (β_std_ = 0.19, p_FDR_ = 0.022), Mini Mental State Exam score (β_std_ = 0.23, p_FDR_ = 0.010) as well as MIND score (β_std_ = 0.20, p_FDR_ = 0.022). The remaining cognitive scores, diet scores and hsCRP showed no significant differences (Supplementary Figure S9). Moreover, brain structural indices and mental health scores also showed no significant differences. Bootstrap regression confirmed robustness of these results (Supplementary Table S4 and S5). These results were stable across models reflecting different sequential adjustment steps (Supplementary Figure S10).

### Enrichment ratios and group assignments are robust to different parameter choices

To evaluate the potential influence of pipeline design choices on our findings, we reanalysed the data using 17 different pipeline configurations, varying the components and parameters of the topological data analysis (Supplementary Table S6). The results demonstrated high robustness, with Spearman correlations between enrichment ratios from the original and alternative configurations averaging 0.82 ± 0.03. Furthermore, the ARI for group assignments derived from k-means clustering via the original pipeline, compared to those from different configurations, remained consistently high at 0.84 ± 0.06.

## Discussion

In this population-based study, we integrated subgingival microbiota profiles with a broad set of brain health phenotypes and identified a continuous pathogenicity gradient that captured interindividual differences in microbial composition. Along this gradient, higher relative abundance of periodontitis-associated taxa was linked to poorer cognitive performance, elevated leucocyte counts, and lower adherence to the MIND diet, even after accounting for demographic and cardiovascular risk factors. These results were supported by a forward model selection analysis, which independently confirmed significant associations between the microbiota and cognitive performance, systemic inflammation, and brain structure. Our findings highlight several key insights into the oral microbiome–brain axis, potential pathophysiological pathways and clinical implications.

### Topological data analysis reveals a latent axis of periodontitis-related microbial composition

We performed a dominance analysis to identify bacterial genera enriched within clusters of the microbiota similarity network. This revealed a gradient in microbial composition from periodontitis-associated taxa to health-associated taxa (Fig. 3b). Genera linked to periodontal disease—such as *Porphyromonas*, *Fusobacterium*, *Treponema*, *Saccharibacteria* (TM7), *Campylobacter*, and *Dialister*—were enriched at one pole of this gradient, consistent with their established roles in biofilm formation, immune modulation, and periodontal tissue destruction.^7^^,^^46^, ^47^, ^48^, ^49^ In contrast, genera typically associated with oral health or low periodontal pathogenicity, including *Streptococcus*, *Haemophilus*, *Rothia*, *Veillonella*, and *Neisseria*, were enriched at the opposing pole.^50^^,^^51^ The consistently high enrichment ratios confirmed that the network captured meaningful interindividual variation in microbiota composition.

Together, these findings indicate that subgingival microbiota composition in the general population is organised along a biologically interpretable continuum of periodontal pathogenicity. While the microbiota similarity network captures broad population-level trends, it also enables individual-level profiling: a person's specific position within this topological space reflects their unique microbial community and associated clinical traits, such as lower cognitive performance. This evidence demonstrates that a topological data analysis-based approach can uncover subtle yet biologically meaningful patterns of disease severity in large-scale, highly complex datasets.

### Microbial compositions are linked to non-microbiome phenotypes

The microbiota similarity network provides a framework for integrating high-dimensional microbiome data with detailed health information by mapping non-microbial phenotypes onto the network and testing their alignment with its structure. Phenotypes showed varying alignment with the network structure (Fig. 4b), ranging from strong, linear associations along the pathogenicity gradient (e.g., leukocytes) to weaker or non-linear patterns (e.g., PHQ-9). Co-enrichment analyses further supported these findings: microbial and non-microbial phenotypes with visually similar enrichment patterns also clustered together in principal component space and showed strong correlations in their enrichment scores (Figs. 5 and 6; Supplementary Figures S5–S7).

To compare analytical approaches, we examined the relationship between the SAFE enrichment ratio and variance explained by alternative methods. The enrichment ratio correlated strongly with adjusted R^2^ values from adonis (ρ = 0.83) and envfit (ρ = 0.79) which quantify the variance in subgingival microbiota explained by host phenotypes. This indicates consistency between the topological framework and conventional effect-size estimates. Although variable rankings differed slightly, we argue that this reflects complementary sensitivities (Supplementary Figure S3): the topological approach captures nuanced or non-linear patterns, whereas traditional analyses aid comparison with previous work.

Our analyses identified links of microbiota composition to broader host systemic and brain health-related measures. Among these, periodontitis indicators, including plaque index, clinical attachment loss, and bleeding on probing, showed the strongest alignment with the pathogenicity gradient and were also selected as leading contributors to microbiota variation in the forward model analysis (Supplementary Figure S4). These findings underscore that the network primarily reflects periodontal pathology and that genus-level relative abundance profiles capture salient clinical and behavioural information.^48^^,^^52^ Demographic factors such as sex and education level also showed significant associations with microbiota configuration. These variables likely reflect broader socio-behavioural determinants of oral health, as lower educational attainment and sex-related differences in health behaviours have previously been linked to periodontal disease risk and oral hygiene practices. Overall, host phenotypes explained 4.29% of variance in microbial composition, consistent with estimates from other large population studies and indicative of modest but measurable links between host and the subgingival microbiota.^53^^,^^54^

### Periodontal dysbiosis and brain health

The mechanisms linking periodontal dysbiosis to brain health are multifaceted and remain incompletely understood. By integrating microbiome, cognitive, inflammatory, and neuroimaging phenotypes within a unified framework, our findings provide converging evidence of the oral–brain axis in a population-based cohort.

A central feature of our results is the co-alignment of microbial composition, cognitive performance, and systemic inflammation along a continuous pathogenicity gradient (Figs. 4 and 5). Individuals positioned at the pathogenic end of the microbiota network exhibited higher leucocyte levels alongside poorer cognitive performance (Fig. 7). These observations extend prior findings from clinical dementia cohorts to a predominantly non-demented population^12^^,^^55^ and align with earlier studies linking periodontal disease severity to cognitive impairment.^56^^,^^57^

They further support the hypothesis that chronic oral infections may contribute to cognitive decline through immune-mediated pathways, consistent with inflammatory models of neurodegeneration in which sustained peripheral immune activation contributes to central nervous system dysfunction.^4^^,^^10^ Assessment of additional inflammatory markers, such as differential leucocyte counts, interleukins (ILs), and tumour necrosis factor α (TNF-α), could provide complementary insights into the underlying mechanisms.

At the taxonomic level, several genera previously implicated in Alzheimer's disease—including *Porphyromonas*, *Treponema*, *Fusobacterium*, and *Prevotella*—showed abundance patterns consistent with established experimental and clinical evidence (Fig. 7b and c). *P. gingivalis* promotes neurodegenerative processes in animal models, and its presence in human brain tissue is linked to elevated Alzheimer's disease risk.^9^^,^^10^^,^^58^ *T. denticola* induces tau hyperphosphorylation and neuroinflammatory responses,^59^ while antibody levels against *Prevotella intermedia* and *Fusobacterium nucleatum* are higher in patients with AD^60^; these taxa have also been associated with APOE4 status and cerebrovascular lesions.^11^^,^^61^ We also identified genera not previously linked to brain health, including *Fretibacterium*, *Tannerella*, *Dialister*, *Parvimonas*, *Filifactor*, *Peptostreptococcaceae*, and *Mogibacterium*, indicating the need for further study to determine whether they independently influence cognitive decline or co-occur with established oral pathogens.

While our cross-sectional design precludes causal inference, the convergence of microbial, inflammatory, brain structural, and cognitive associative findings within a single cohort supports the notion that oral dysbiosis may be linked to brain health through systemic pathways. Several interconnected biological mechanisms may underlie the observed associations.

At the oral level, a shift toward periodontitis-associated taxa promotes chronic gingival inflammation, reflected in the co-enrichment of clinical attachment loss, bleeding on probing, and periodontal inflamed surface area with pathogenic microbial profiles. This local inflammatory state can propagate systemically via recurrent bacteraemia and increased circulating pro-inflammatory factors, including IL-1β, IL-6, and TNF-α.^10^ In parallel, periodontal pathogens and their virulence factors—such as lipopolysaccharides, outer membrane vesicles, and proteolytic enzymes like gingipains—may enter the circulation and reach the central nervous system.^10^

These processes can compromise blood–brain barrier integrity and promote neuroinflammation. For example, *P. gingivalis* gingipains have been shown to degrade endothelial junction proteins such as PECAM-1 and VE-cadherin,^10^ increasing barrier permeability, while also exerting direct proteolytic effects on proteins central to neurodegeneration, including tau and amyloid-β.^9^^,^^10^^,^^58^ In addition, circulating inflammatory mediators can activate microglia, sustaining a chronic neuroinflammatory environment.^62^

Downstream, these mechanisms converge on neurodegenerative processes, consistent with our finding that cortical thickness and subcortical volume were associated with microbiota composition in forward model selection. This finding may reflect subtle or spatially heterogeneous effects not captured by global summary metrics. Future studies using regionally resolved or voxel-wise analyses will be important to clarify the anatomical specificity of these associations.

At the same time, reverse causality remains a plausible explanation, as reduced cognitive function may impair oral hygiene and thereby promote periodontal dysbiosis. Longitudinal and interventional studies will be required to disentangle these bidirectional relationships and to determine whether modifying the oral microbiome can alter trajectories of cognitive decline.

### Subgingival microbiota and mental health

In contrast to cognitive measures, most mental health associations were weaker and lost significance after adjustment. A notable exception was somatic symptom severity (PHQ-15), which was retained as a significant contributor in the forward model selection analysis. This complements prior evidence on associations among anxiety, depression, and periodontal disease,^63^ and suggests that somatic symptoms may represent an additional dimension of the oral microbiota–mental health relationship.

### Influence of diet and vascular risk factors on oral and brain health

Diet was a key correlate of subgingival microbiota composition. Individuals enriched for periodontitis-associated taxa reported poorer adherence to cognitively beneficial diets (MEDAS, MIND, DASH), and after covariate adjustment, lower MIND diet adherence remained significantly associated with a more pathogenic profile. These results align with evidence that anti-inflammatory diets reduce periodontitis risk^64^ and suggest that diets promoting cognitive health may also support a healthier oral microbiome, potentially through nutrient-driven and immune-mediated mechanisms.^65^ Longitudinal and interventional studies are needed to determine causal pathways linking diet, microbial ecology, and cognitive outcomes.

Among vascular and demographic factors, smoking was the only variable relevantly associated with the pathogenicity gradient. Smoking is known to promote colonisation by periodontal pathogens, enhance biofilm formation, and impair immune responses.^66^ Our results reinforce its role as a key driver of shifts in microbial composition, inflammation, and associated brain health phenotypes.

### Clinical implications

Our findings help elucidate systemic pathways linking periodontal dysbiosis to brain health and highlight potential clinical applications. Subgingival microbiota composition was associated with variation in cognitive performance and brain structure, suggesting possible utility in early risk assessment for cognitive decline. Identifying shifts in microbial communities before cognitive symptoms emerge may complement existing dementia biomarkers and support targeted recruitment for early-intervention trials.

Microbiome signatures of early periodontal dysbiosis may also inform the development of oral microbiome–directed therapies aimed at slowing or preventing neurodegenerative processes.^67^ However, the role of microbiome-based markers in cognitive disorders remains to be established, and large longitudinal and interventional studies will be required before clinical translation is feasible.

### Strengths and limitations

Strengths of this study include the large population-based cohort, comprehensive microbiota profiling based on subgingival plaque samples, and the integration of clinical, lifestyle, and neuroimaging data via a unified topology-based framework. Specifically, our study extends the current literature in three key ways: First, subgingival plaque sampling enables targeted profiling of the periodontal pocket microbiota—typically anaerobic and proteolytic organisms that may not be adequately captured by oral rinse samples commonly used in previous studies. Second, the extensive phenotyping of brain health, including cognitive testing, structural MRI, inflammatory biomarkers, dietary patterns, and vascular risk factors, allows a multidimensional assessment of the oral microbiome–brain health axis within a non-demented population. Third, our topology-based microbiota similarity network captures continuous microbial community gradients from health-associated to periodontitis-associated profiles, enabling systematic integration of community structure with multiple brain health phenotypes simultaneously—complementing conventional taxon-by-taxon approaches.

However, the primary limitation is the cross-sectional design, which precludes the resolution of temporal associations. Notably, reverse causality cannot be ruled out, as poorer cognition may lead to impaired oral hygiene and consequently to alterations in the subgingival microbiota. Individuals with cognitive decline may neglect their oral health, which could contribute to the higher abundance of periodontitis-associated taxa observed in this study. Both causal directions therefore remain plausible. Another limitation is the single-centre design, as all participants were recruited from the HCHS, an urban German population-based cohort. This may limit external validity, as oral microbiome composition and periodontal disease risk can vary across populations due to differences in ethnicity, dietary habits, healthcare systems, and other environmental or lifestyle factors. Furthermore, participants were cognitively normal individuals aged 45–75 years, reflecting the HCHS recruitment criteria. While this age range is well suited to investigate early mechanisms of cognitive decline, it may limit generalisability beyond this age interval. Additional limitations include potential unmeasured confounders such as relatedness, the use of pooled subgingival samples, and reliance on 16S rRNA gene amplicon sequencing, which restricts functional interpretation. Specifically, analysing the data at the genus level—while necessary to ensure adequate statistical power—potentially masks critical species-level variations. Future longitudinal and metagenomic analyses are required to clarify causal pathways and better characterise microbial strains and functions.

### Conclusion

Using a comprehensive analytical framework, we identified latent associations between periodontitis-related microbial composition and cognitive performance, brain structural measures, systemic inflammation, and dietary patterns in a population-based sample. From a translational perspective, these findings suggest that periodontal microbial profiling may contribute to multimodal risk stratification in cognitive decline and dementia. However, the clinical utility of oral microbiome analyses as a diagnostic tool or therapeutic target remains to be established, requiring validation in patient cohorts, longitudinal studies, and evidence of incremental value beyond established risk markers.

## Contributors

Each author has made a significant contribution to the manuscript and all authors read and approved its final version. We describe contributions to the paper using the CRediT contributor role taxonomy. M.P.: Conceptualisation, Data curation, Formal analysis, Investigation, Methodology, Project administration, Resources, Software, Visualisation, Funding, Writing—original draft, Writing—review & editing; C.W.: Conceptualisation, Data curation, Investigation, Methodology, Resources, Writing—original draft, Writing—review & editing; K.B.: Data curation, Methodology, Investigation, Resources, Software, Writing—review & editing; G.H.: Resources, Writing—review & editing; T.B.: Resources, Writing—review & editing; M.A.: Resources, Writing—review & editing; C.M.: Resources, Writing—review & editing; F.L.N.: Data curation, Resources, Writing—review & editing; B.Z.: Resources, Writing—review & editing; J.F.: Resources, Writing—review & editing; J.G.: Resources, Writing—review & editing; S.K.: Resources, Writing—review & editing; R.T.: Resources, Writing—review & editing; C.B.: Methodology, Writing—review & editing; G.T.: Supervision, Funding, Writing—review & editing; B.C.: Conceptualisation, Funding, Project administration, Resources, Supervision, Writing—original draft, Writing—review & editing; G.A.: Conceptualisation, Funding, Project administration, Resources, Supervision, Writing—original draft, Writing—review & editing. M.P, C.W., B.C., and G.A. have accessed and verified the underlying data.

## Data sharing statement

Sequencing data generated during this study have been deposited as FASTQ files in the European Nucleotide Archive (ENA) under accession number PRJEB89258. Corresponding non-microbiome phenotype data from the HCHS are not publicly available due to data protection policies that ensure participant confidentiality. However, this data is available to qualified researchers upon reasonable request to the HCHS steering committee. The analysis code for this work is publicly available on GitHub (https://github.com/csi-hamburg/oral_microbiome_brain_health). Interactive versions of the plots can be found on OSF (https://osf.io/vqj8m/).

## Declaration of interests

J.G. reports grants from the German Research Foundation (DFG; GRK 2753), the Federal Ministry of Education and Research (BMBF; HIVAM and EnTree, BMBF 16SV9423), the Federal Ministry of Research, Technology and Space (BMFTR; DiaCare VIP+), and the Hamburg City Health Authority (Psychiatrieplan); royalties from Elsevier; consulting fees from Boehringer; and speaker fees from Lundbeck and Boehringer. R.T. reports grants paid to the institution from the German Center for Cardiovascular Research (DZHK), the Kühne Foundation, the Joachim Herz Foundation, the Swiss National Science Foundation (P300PB_167803), and the Swiss Heart Foundation; payment or honoraria from Abbott, Amgen, AstraZeneca, Psyros, Roche, Siemens, Singulex, SpinChip, and Thermo Scientific BRAHMS; advisory board participation for Roche and Amgen; an international patent application (WO2022043229A1, TW202219980A); and being a co-founder and shareholder of ARTEMIS Hamburg GmbH. G.T. reports consulting fees from Acandis, Bayer, Boehringer Ingelheim, and TarGED; payment or honoraria from Acandis, AstraZeneca, Bayer, and Boehringer Ingelheim; and a leadership role as Chair of the Board of Directors of the European Stroke Organisation (ESO). The remaining authors declare no competing interests.
