# Supplementary material for: Subgingival microbiota composition is associated with brain health in the general population–the PAROMIND study
Source: eBioMedicine. 2026 May 30;128:106312. doi: 10.1016/j.ebiom.2026.106312 (PMC13241667; doi:10.1016/j.ebiom.2026.106312)
Supplement: Supplementary Material [file mmc1.pdf]

Supporting Information for

Subgingival microbiota composition is associated with brain health in the general population - the PAROMIND Study

Marvin Petersen, Carolin Walther, Katrin Borof, Guido Heydecke, Thomas Beikler, Malik Alawi, Christian Müller, Felix L. Nägele, Birgit-Christiane Zyriax, Jens Fiehler, Jürgen Gallinat, Simone Kühn, Raphael Twerenbold, Corinna Bang, Götz Thomalla, Bastian Cheng, Ghazal Aarabi

Corresponding authors: Marvin Petersen (mar.petersen@uke.de) & Ghazal Aarabi (g.aarabi@uke.de)

This PDF file includes:

Supplementary Text – Supplementary Methods
Figs. S1 to S10
Table S1 to S6
References (1 to 48)

Supplementary Text

Supplementary methods

Our methodology, which integrates microbiome, clinical and lifestyle data into a unified analysis framework, is illustrated in *figure 1a* and *1b*.

Study design

This analysis integrates different data domains related to oral and brain health into a unified analysis framework: subgingival microbiota composition, oral health status, cognitive function, mental health status, brain structure, circulating inflammatory markers, dietary patterns, vascular risk and demographics. In brief, we employed topological data analysis, specifically the Mapper algorithm, to create a low-dimensional network representation of subgingival microbiota data [1]. This network positions individuals based on similarities in their subgingival microbiota composition. Subsequently, we conducted an enrichment analysis using Spatial Analysis of Functional Enrichment (SAFE) [2]. This analysis statistically assesses regions of the network derived via Mapper to identify where specific phenotypes are significantly higher or lower than expected by chance. This approach allowed us to examine regional differences in phenotypes, revealing whether the network representation captures variance in specific participant traits related to oral and brain health. Finally, we performed a secondary group comparison between participants of two distinct participant clusters within the microbiota network, enabling us to assess the link of subgingival microbiota composition and clinical and lifestyle phenotypes while adjusting for relevant covariates.

Molecular analysis and phenotyping of the oral microbiota

Oral microbiota phenotyping followed a standardized procedure targeting the subgingival environment within periodontal pockets. This specific environment was chosen because it contains the subgingival biofilms most directly implicated in periodontitis pathogenesis, distinguishing its microbial community from those in other oral sites like saliva or supragingival plaque. During the dental examination, gingival crevicular fluid samples were collected from periodontal pockets with sterile paper points. Two samples per participant were obtained: [1] from a single deep periodontal pocket and [2] one pooled sample (4 paper points) from the deepest periodontal pockets. Each paper point remained in situ for 15 seconds. Subsequently, samples were placed in a sterile 2ml-Eppendorf tube and stored at -80°C in the HCHS biobank. For the following molecular analysis, only pooled samples were further processed.

Sequencing was performed at the Institute of Clinical and Molecular Biology in Kiel, Germany. All samples were processed with negative and positive controls. The amount of DNA per sample was quantified prior to amplification to ensure it met the required minimum of 50 ng, which is particularly important for low-biomass samples like gingival crevicular fluid. Bacterial composition was determined via 16S rRNA gene amplicon sequencing (Illumina). DNA Isolation was performed using the DNA extraction kit from Innuprep Analytics (Analytik Jena AG, Überlingen, Germany). For the initial lysis, samples were incubated in Tris-EDTA buffer with lysozyme (10 mg/ml stock solution) at 37 °C until lysis was achieved. Subsequently, lysis solution TLS and Proteinase K were added, followed by incubation at 50 °C for 15 minutes. For the sequencing,

variable regions V3 and V4 of the 16S rRNA gene were amplified using the primer pair 341F (5‘-CCTACGGGAGGCAGCAG-3‘) and 806R (5‘-GGACTACHVGGGTWTCTAAT-3’) in a dual barcoding approach [3,4]. Briefly, 26 µl PCR mix comprising 5.0 µl 5X Phusion HF buffer, 0.5 µl dNTP (10 mM), 0.3 µl Phusion Hot Start II Polymerase (2 U/µl; Thermo Fisher Scientific), 9.2 H2O, 4 µl of each uniquely barcoded 16S primer (100 µM), and 3 µl microbial DNA template were prepared. Negative controls (nuclease-free water) as well as positive controls (ZymoBIOMICS Microbial Community Standard, Cat. no. D6305) were included on each PCR reaction plate. Following an initial denaturation temperature of 98 °C for 30 s, 30 PCR cycles were carried out with 98 °C (0:09 min), 55 °C (1:00 min) annealing temperature, and 72 °C (1:30 min) extension temperature as well as a final round at 72 °C for 10:00 min. Agarose gel electrophoresis was used to verify resulting PCR products (~550 bp) before normalization using the SequalPrep Normalization Plate Kit (Thermo Fischer Scientific, Waltham, MA, USA), pooling and sequencing on the Illumina MiSeq with v3 2x300bp chemistry (Illumina Inc., San Diego, CA, USA). Demultiplexing after sequencing was based on 0 mismatches in the barcode sequences.

Data processing was performed using the DADA2 workflow for big datasets (v. 1.10.42, https://benjjneb.github.io/dada2/bigdata.html), resulting in relative abundance tables of amplicon sequence variants (ASVs). For this, all sequencing runs were handled separately and finally collected in a single relative abundance table per dataset, which underwent chimera filtering. ASVs underwent taxonomic annotation using the Bayesian classifier provided in DADA2 and using the expanded Human Oral Microbiome Project (eHOMD) version V15.23. Samples with less than 10,000 sequences were not considered for further analysis. Downstream analyses were performed at the genus-level because this taxonomic rank provided a suitable balance, enhancing statistical power across the large cohort, improving interpretability and comparability with existing literature, and reducing data dimensionality while still providing sufficient resolution for the study's objectives. Genera that were observed with a frequency of less than 0.1% of all genera detected in a sample were discarded.

Oral health assessment

A certified study nurse assessed probing depth and gingival recession at six sites of the tooth (mesio-buccal, buccal, disto-buccal, disto-palatinal, palatinal and mesio-palatinal). Study nurses were trained and certified by licensed dentists experienced in periodontal examination procedures prior to data collection. The plaque index (PI) was measured at two interproximal sites per tooth to evaluate dental plaque accumulation. Clinical attachment loss (CAL) was calculated as the sum of probing depth and gingival recession [5]. The BOP index was determined at two sites per tooth (vestibular, oral) and expressed as a percentage of bleeding sites. All measurements were completed using a standard periodontal probe (PCP 15, Hu-Friedy, Chicago, IL, USA). The DMFT index was recorded. The PISA was calculated with a custom-made R function, based on a pre-existing and freely available Excel spreadsheet (https://www.parsprototo.info/); the formula was adjusted for the two-site BOP protocol used in the HCHS. The oral health assessment followed a standardized protocol for the reporting of the prevalence and severity of periodontal diseases [6]. Periodontitis staging was determined according to the “2017 AAP/EFP periodontitis classification”, applying the framework for use in epidemiological data (“Application of the 2018 periodontal status Classification to Epidemiological Survey data”; ACES) [7–9]. Accordingly, study participants were categorized into edentulism, periodontal health, gingivitis, non-classified and

periodontitis. Periodontitis cases were subsequently staged into Stage I, Stage II, Stage III and Stage IV [9].

Cognitive and mental health assessments

Cognitive testing was conducted using the extended version of the Consortium to Establish a Registry for Alzheimer's Disease Neuropsychological Assessment Battery (CERAD-NP/Plus) [10]. A trained study nurse administered all tests. For this analysis, we considered cognitive scores measuring executive function (Trail Making Test B), information processing speed (Trail Making Test A), memory (Word List Recall Test), reasoning (Multiple Choice Vocabulary Intelligence Test B), verbal fluency (Animal Naming Test), and the Mini Mental State Exam. To ensure higher scores indicated better cognitive performance across all tests, we inverted the results of Trail Making Test A and B. Subsequently, we performed a principal component analysis (PCA) on all individual test scores. Following previous procedures, the first principal component, which accounted for the greatest variance (40.5%), was defined as a measure of general cognitive ability (g) (for details see *supplementary figure S1*) [11]. According to the principal component loadings, higher values of this measure corresponded to lower cognitive performance; thus, it was also inverted. Furthermore, participants underwent mental health assessments via established questionnaires for depression (PHQ-9, Geriatric Depression Scale), somatic symptom severity (PHQ-15), and anxiety (GAD-7) [12–14].

Neuroimaging of brain macro- and microstructure

Neuroimaging markers representing different aspects of macro- and microstructural brain integrity were computed based on T1-weighted and diffusion-weighted MRI following previous procedures [15]. After cortical surface reconstruction and subcortical segmentation based on T1-weighted images with FreeSurfer, cortical thickness and subcortical volume were estimated representing morphometric measures of neurodegenerative processes [16]. The mean cortical thickness and mean subcortical volume were z-scored. Subsequently, the two resulting z scores were averaged to obtain a single summary measure reflecting cortical thickness and subcortical volume. Following preprocessing of the diffusion-weighted images, free-water imaging was employed to compute free-water which quantifies the amount of extracellular water as well as tissue fractional anisotropy reflecting neurite architecture and integrity [17]. The free-water and tissue fractional anisotropy values were then averaged across cortical and subcortical gray matter voxels, as well as white matter voxels, to obtain global measures of gray and white matter, respectively. In the following sections, we provide a detailed account of the acquisition protocol, quality assessment, preprocessing and computation procedures on the different imaging measures.

<u>Brain MRI acquisition</u>

MR imaging was conducted using a single 3-Tesla Siemens Skyra. The scanning protocols follow those outlined in previous studies. For 3D T1-weighted anatomical images, a rapid acquisition gradient-echo sequence (MPRAGE) was used with the following sequence specifics: repetition time (TR) = 2500 ms, echo time (TE) = 2.12 ms, 256 axial slices, slice thickness (ST) = 0.94 mm, in-plane resolution (IPR) = 0.83 × 0.83 mm. 3D T2-weighted fluid-attenuated inversion recovery (FLAIR) images with TR = 4700 ms, TE = 392 ms, 192 axial slices, ST = 0.9 mm, and IPR = 0.75 × 0.75 mm. For single-shell diffusion-weighted imaging (DWI) 75 axial slices were acquired with

gradients (b = 1000 s/mm^2) applied along 64 noncollinear directions with the following sequence parameters: TR = 8500 ms, TE = 75 ms, ST = 2 mm, IPR = 2 × 2 mm, anterior–posterior phase-encoding direction, 1 b0 volume.

Preprocessing of brain MRI data

MRI data were processed employing preconfigured containerized pipelines as described previously [15]. The full documentation can be found on GitHub (https://github.com/csi-hamburg/CSIframe/wiki). Diffusion- and T1-weighted images were preprocessed employing QSIprep (v. 0.14.2) [18]. DWI preprocessing included bias field correction, skull-stripping, denoising, deringing, as well as correction of B1 field inhomogeneity, head motion, eddy currents, and susceptibility distortions [18–22]. T1-weighted and FLAIR images underwent bias field correction and skull-stripping [22].

Quality analysis of neuroimaging data

Quality assessment included visual inspection of raw images and processed images based on quantitative outliers defined as measures exceeding 2 standard deviations from the mean of quality measures computed via QSIprep and mriqc [18,23].

Computation of cortical thickness and volume.

FreeSurfer (v. 6.0.1) was used to derive regional measures of cortical thickness and subcortical volumes [24,25]. After surface reconstruction, cortical thickness was measured as the distance between the white matter and pial surface [25]. Cortical thickness was averaged across the cortex. Subcortical volumes were averaged across all structures of the aseg atlas [24]. The mean cortical thickness and mean subcortical volume were z-scored. Subsequently, the two resulting z scores were averaged to obtain a single summary measure of cortical thickness and subcortical volume.

Computation of free-water and tissue fractional anisotropy.

Diffusion imaging features were derived from preprocessed diffusion-weighted images. We conducted free-water imaging, employing a dual-tensor model that delineates the isotropic extracellular compartment and the cellular compartment characterized by hindered or restricted diffusion. Based on a regularized non-linear fitting process, free-water and tissue fractional anisotropy (FA_T) were calculated [26]. Voxel-level free-water and tissue fractional anisotropy were averaged across gray matter voxels as well as across white matter voxels to obtain global measures for gray and white matter, respectively.

Diet
The dietary behavior of all participants was assessed using a validated food frequency questionnaire with 102 items, developed for the European Prospective Investigation into Cancer and Nutrition Study (EPIC) [27]. The adherence to different dietary patterns was measured based on the food frequency questionnaire scores, including the Mediterranean diet (MEDAS diet), the Dietary Approaches to Stop Hypertension (DASH diet), and the Mediterranean-DASH Intervention for Neurodegenerative Delay (MIND diet) [28–30]. Adherence to the Mediterranean diet was determined using the German version of the Mediterranean Diet Adherence Screener, which assigns a score of 0 or 1 to 14 specific food items, producing a total adherence score ranging from 0 (no adherence) to 14 (maximum adherence) [28]. The DASH diet was evaluated using a previously established scoring method that assigns a score of 0, 0.5, or 1 to each of 10 items, resulting in an adherence score between 0 (no adherence) and 10 (maximum adherence) [29]. Finally, adherence to the MIND diet was calculated according to standard procedures: scores of 0, 0.5, or 1 to are assigned 10 healthy and 5 unhealthy food items, culminating in a total adherence score ranging from 0 (no adherence) to 15 (maximum adherence) [30].

Topological data analysis
We implemented a topological data analysis pipeline that integrates two key components: [1] the Mapper algorithm, which performs an unsupervised reconstruction of a topological network based on genus-level relative abundance data, capturing microbiota composition similarity, and [2] SAFE, which conducts statistical tests to examine the relationship between the network's structure and different phenotypes [1,2]. This method effectively combines dimensionality reduction with topological insights, offering a powerful tool for understanding the intrinsic geometry of high-dimensional microbiota data and its relationships with other data domains. The analysis was performed in *python* v3.8.1 based on the packages *NetworkX* v2.2 (https://github.com/networkx/networkx), *safepy* (https://github.com/baryshnikova-lab/safepy), *scikit-learn* v1.5.1 (https://github.com/scikit-learn/scikit-learn) and *tmap* v1.2 (https://github.com/GPZ-Bioinfo/tmap) as well as *R* v4.4.0 based on the package *vegan v2.6-6.1* [1,2]. Data visualization was based on *plotly* v5.22 (https://github.com/plotly/plotly.py) and iTOL v6 (https://itol.embl.de/). HTML versions of many presented plots can be found on OSF allowing interactive data exploration (https://osf.io/vqj8m/).

Mapper: Reconstruction of the microbiota similarity network
Genus-level subgingival microbiota relative abundance data served as input to the Mapper algorithm, a topological data analysis technique that simplifies complex high-dimensional data by constructing a topological network capturing essential relationships and patterns in the data. This network preserves the data's underlying topological and geometric structure by positioning participants with similar subgingival microbiota profiles nearby. Conceptually, this representation is analogous to a topographical map that reveals the essential features of a landscape. Importantly, the network can represent non-linear associations that conventional linear techniques might miss. Mapper has previously been used to analyze the dynamic organization of brain function [31,32], the shape of genetic data in breast-cancer patients [33], biomolecular folding pathways [34], brain structure in patients with fragile X syndrome [35], and neuronal data from the visual cortex [36].

The applied Mapper pipeline comprises multiple analysis steps to reconstruct the topological network (for an illustration see *figure 1b*): filtering, covering, clustering and network reconstruction [37]. First, as the filtering step, we conducted a principal coordinate analysis of the Aitchison distance of genus-level relative abundance ($n_{participants}$ x n_{genera}) [38]. To account for the compositional nature of the data and handle zero values, the distance was calculated using the robust Aitchison method [39]. By that, we obtained two components – also called lenses – capturing the major axes of variation in the microbial community composition across the participants. Based on these axes, overlapping covers were defined (overlap = 1.5, resolution = 30) to segment the data into overlapping bins, each representing a local region of inter-individual variation. Unsupervised clustering of datapoints within each bin was performed using Hierarchical Density-Based Spatial Clustering of Applications with Noise (HDBSCAN, epsilon threshold = 0.95) [40]. By this, nodes are obtained that represent participant groups with similar configuration of the subgingival microbiota. Participants can belong to multiple nodes with the number varying per participant. Lastly, the network reconstruction was accomplished by connecting clusters sharing common participants. Not all datapoints are retained by Mapper resulting in the omission of some participants ($n_{not\ retained}$ = 109).

SAFE: Enrichment analysis

SAFE (Spatial Analysis of Functional Enrichment) is a network annotation method originally developed for genetic interaction networks [2]. The core idea is that if a biological attribute – such as a clinical phenotype or microbial abundance – is non-randomly distributed across a network, spatially concentrated regions of high or low values can be detected via permutation testing. Unlike conventional association tests that operate on individual-level data, SAFE exploits the topology of the network itself: it asks whether the values of a given attribute are more spatially clustered within the network than expected by chance, thereby linking network structure to biological function.

In our work, we performed SAFE on the microbiota similarity network derived using the Mapper algorithm to identify regions within the network that are significantly enriched for specific participant traits. Specifically, we investigated the enrichment of genus-level relative abundance, oral health measures (clinical attachment loss, plaque index, bleeding on probing index, DMFT index, PISA, missing teeth, ACES classification), cognitive scores (general cognitive ability, Animal Naming Test, Mini Mental State Exam, Multiple Choice Vocabulary Intelligence Test B, Trail Making Tests A and B, Word List Recall), mental health scores (PHQ-9, PHQ-15, Geriatric Depression Scale, GAD-7), imaging measures (cortical thickness and subcortical volume, gray matter free-water, white matter free-water, gray matter tissue fractional anisotropy, white matter tissue fractional anisotropy), circulating inflammatory markers (hsCRP, leukocytes), diet scores (MEDAS score, DASH score, MIND score), vascular risk factors (systolic and diastolic blood pressure, body mass index, smoking behavior, blood triglycerides, cholesterol, low density lipoprotein, high density lipoprotein, HbA1c), and demographics (age, sex, education).

Following previous analyses leveraging SAFE [2,41], the microbiota similarity network was spring-embedded, i.e., nodes in the network were positioned so that those with connections are placed

closer together, while repulsive forces push non-connected nodes apart, resulting in a visually balanced and interpretable representation of the network's structure [42]. Next, we computed node attributes for all phenotypes by averaging the values of each variable across all participants within a node. Subsequently, enrichment scores were calculated for each node following a four-step process (*figure 1c*). First, we defined the local neighborhood by identifying all nodes within a maximum distance threshold of 0.75 from the central node. The distance was measured using the map-weighted shortest path length (MSPL) [2]. Second, we calculated a neighborhood score by summing the attribute values of neighboring nodes. Third, we computed a p-value by comparing the empirical neighborhood score against a distribution derived from 5000 permutations. Permutations were performed by randomly reassigning attributes to nodes while preserving the network topology [2]. The resulting p-value was corrected for multiple comparisons across all phenotypes. Finally, we assigned an enrichment score to the neighborhood center by applying a -log10 transformation to the corrected p-value. Given the 5000 permutations, the maximal enrichment score is -log10(1/5000) = 3.70, with -log10(0.05) = 1.30 indicating significance. Positive enrichment scores indicate that observed values are higher than the permuted distribution, negative enrichment scores indicate that they are lower than the permuted distribution. This procedure is repeated for each node of the network, resulting in an enrichment map indicating where attributes are higher or lower than expected by chance.

To understand the primary taxonomic patterns shaping the network topology, we performed a dominance analysis alongside examining individual phenotype enrichments. This involved labeling each network node by the single bacterial genus with the highest positive enrichment score. Visualizing this node-level dominance helps to reveal major taxonomic transitions across the network. While highlighting the most strongly enriched genus in each region provides valuable pointers to key drivers, this is a simplification; each node still represents a complex microbial community, not just the dominant genus identified.

Statistical assessment of microbiome-host associations

Our statistical analysis followed a two-stage design. In the primary stage, we used the unsupervised microbiome-derived network topology to examine which brain health-related phenotypes co-vary with microbiome structure, without adjustment for covariates – a deliberate design choice, as the network topology is constructed exclusively from microbiome data, and the phenotypes of interest are themselves outcome variables under investigation. In the secondary stage, we performed a covariate-adjusted group comparison to formally test associations between microbiome-derived group membership and non-microbiome phenotypes (see Group analysis below).

To measure how strongly the microbiota similarity network reflects a specific phenotype we computed the enrichment ratio as the number of significantly enriched nodes (corrected $p < 0.05$) divided by the total number of nodes. A higher enrichment ratio indicates that more nodes are significantly enriched, suggesting that the network's topology captures a greater extent of a phenotype's variance.

While the enrichment ratio quantifies the scope of a phenotype's association across the network's topology, it is not a direct measure of effect size. To provide a complementary, conventional assessment of effect size, we also tested the association between non-microbiome phenotypes and the overall microbiota composition using two statistical methods from the vegan package: 1) envfit ($n_{permutations}$ = 5000) testing the linear association between each phenotype and the principal coordinate analysis (PCoA) ordination (all components) of the robust Aitchison distance matrix; 2) permutational multivariate analysis of variance (PERMANOVA, adonis, $n_{permutations}$ = 5000) testing the proportion of variance in the robust Aitchison distance matrix explained by each phenotype individually. All resulting p-values were false discovery rate-corrected. This complementary analysis was only performed for non-microbiome phenotypes and not for the relative abundance of individual genera.

To quantify the relative contribution of non-microbiome phenotypes while accounting for their overlapping effects, we performed forward model selection using distance-based redundancy analysis (db-RDA, capscale). The analysis was conducted with the ordiR2step function ($n_{permutations}$ = 5000) from *vegan*, using the robust Aitchison distance matrix of the microbiome data as the response variable. Because ordiR2step requires a complete dataset, missing values in the non-microbiome data were imputed using k-nearest neighbors (KNN, $n_{neighbors}$ = 5) prior to model fitting. This imputation was based only on other non-microbiome phenotypes to prevent information leakage from the microbiome data. The procedure sequentially added explanatory variables, retaining only those that significantly improved the model's fit ($p < 0.05$ based on 5000 permutations). The adjusted R2 value for each step was used to quantify the variance explained by each selected variable.

To determine whether specific phenotypes co-enrich, i.e., exhibit similar enrichment patterns, we performed an ordination of the enrichment scores using principal component analysis retaining the first two principal components and assessed the pairwise Spearman correlation between the enrichment scores.

Group analysis

To formally test whether the associations between microbiome-derived group membership and brain health phenotypes are independent of potential confounders we performed a secondary group analysis with covariate adjustment. This analysis constitutes the confirmatory stage of our two-stage framework, in which the microbiome-derived group assignment (determined solely by microbiome topology, without reference to any phenotype) serves as the independent variable, and non-microbiome phenotypes serve as dependent variables in multiple linear regression models adjusting for relevant covariates.

To define groups, nodes of the microbiota similarity network were clustered in two non-overlapping groups using k-Means clustering of the node positions. We chose k-Means as it is widely used and arguably represents the simplest unsupervised clustering technique [43]. k-Means requires to predefine the number of clusters to assign datapoints to. Given that enrichment analysis indicated that most participant traits varied along the microbiota similarity network in a

linear left-right trajectory, i.e., participants on the left end differed from those on the right end, the number of clusters was set to k = 2.

After the clustering, participants were categorized based on the resulting groupings. Importantly, individuals present in both groups – due to being assigned to nodes in both groups – were excluded from the analysis (n = 137). This exclusion was a strict methodological requirement to ensure full statistical independence between the two groups for the subsequent comparative regression models. Biologically, these individuals do not represent a distinct pathological cohort, but rather reflect the intermediate 'transition zone' along the continuous microbial pathogenicity gradient. Given that not all datapoints are retained by the Mapper algorithm during the network reconstruction step, participants that were not represented in the microbiota similarity network were not considered for this analysis. Before the group comparison, a center log-ratio (CLR) transformation was applied to genus-level relative abundance data. The groups were statistically compared for the phenotypes using multiple linear regression and age, sex and education and vascular risk factors (systolic and diastolic blood pressure, body mass index, smoking behavior, triglycerides, cholesterol, LDL, HDL, HbA1c) were included as covariates:

$$Phenotype \sim Group + Age + Sex + Education + Vascular\ risk\ factors$$

Covariates were selected a priori based on literature demonstrating their potential influence on both oral health/microbiome and brain health indices [44–47]. In addition to this fully adjusted model, we performed an exploratory analysis by building models that sequentially added each covariate, allowing us to map the specific influence of each confounder on the primary associations.

Multiple comparisons. SAFE enrichment p-values were FDR-corrected (Benjamini–Hochberg) within each phenotype across all network nodes. Envfit and PERMANOVA p-values were FDR-corrected globally across all tested non-microbiome phenotypes. In the db-RDA forward model selection, the permutation-based stopping criterion ($p < 0.05$) and the adjusted R^2 ceiling of the global model jointly control model complexity, following Blanchet et al. [48]; no additional post-hoc correction was applied. In the group analysis, regression p-values were FDR-corrected across all tested phenotypes within each comparison set (genus-level abundances; non-microbiome phenotypes). All corrections used an FDR threshold of 0.05.

Sensitivity analysis

The parameters and components of our analysis pipeline were selected based on established practices, including strategies to optimize sample coverage as described in the tmap documentation, or utilized default software settings. Recognizing that pipeline parameters can influence resulting network characteristics (e.g., node count, connectivity), we conducted a sensitivity analysis using alternative configurations to verify that our results were not biased by these initial design choices. We systematically explored variations in parameter values and pipeline components, altering the Mapper cover overlap from the original 1.5 to 1, 1.2, 1.4, 1.6, 1.8 and 2; the Mapper cover resolution from 30 to 20, 25, 35, 40 and 45; and the Mapper epsilon threshold from 0.95 to 0.99, and 0.90. Additionally, we adjusted the SAFE distance threshold from 0.75 to 0.5 and 0.99, and the SAFE neighborhood radius from 0.1 to 0.05 and 0.15. We adjusted one parameter from the original pipeline per iteration, resulting in a total of 17 iterations. The

robustness of our results was evaluated by comparing the findings from the original configuration to those from alternative setups. Therefore, we calculated the Spearman correlation of enrichment ratios for clinical and microbiome phenotypes and the Adjusted Rand Index (ARI; ranges from 0 indicating no agreement to 1 indicating full agreement) for group assignments from k-Means clustering to assess stability.

To confirm the robustness of the group comparison results, we performed case-resampling bootstrap regression (10,000 iterations) to estimate the sampling distribution of coefficients empirically, with bias-corrected and accelerated (BCa) 95% confidence intervals. Bootstrap p-values were computed as twice the proportion of bootstrap coefficients on the opposite side of zero from the observed estimate.

Fig. S1

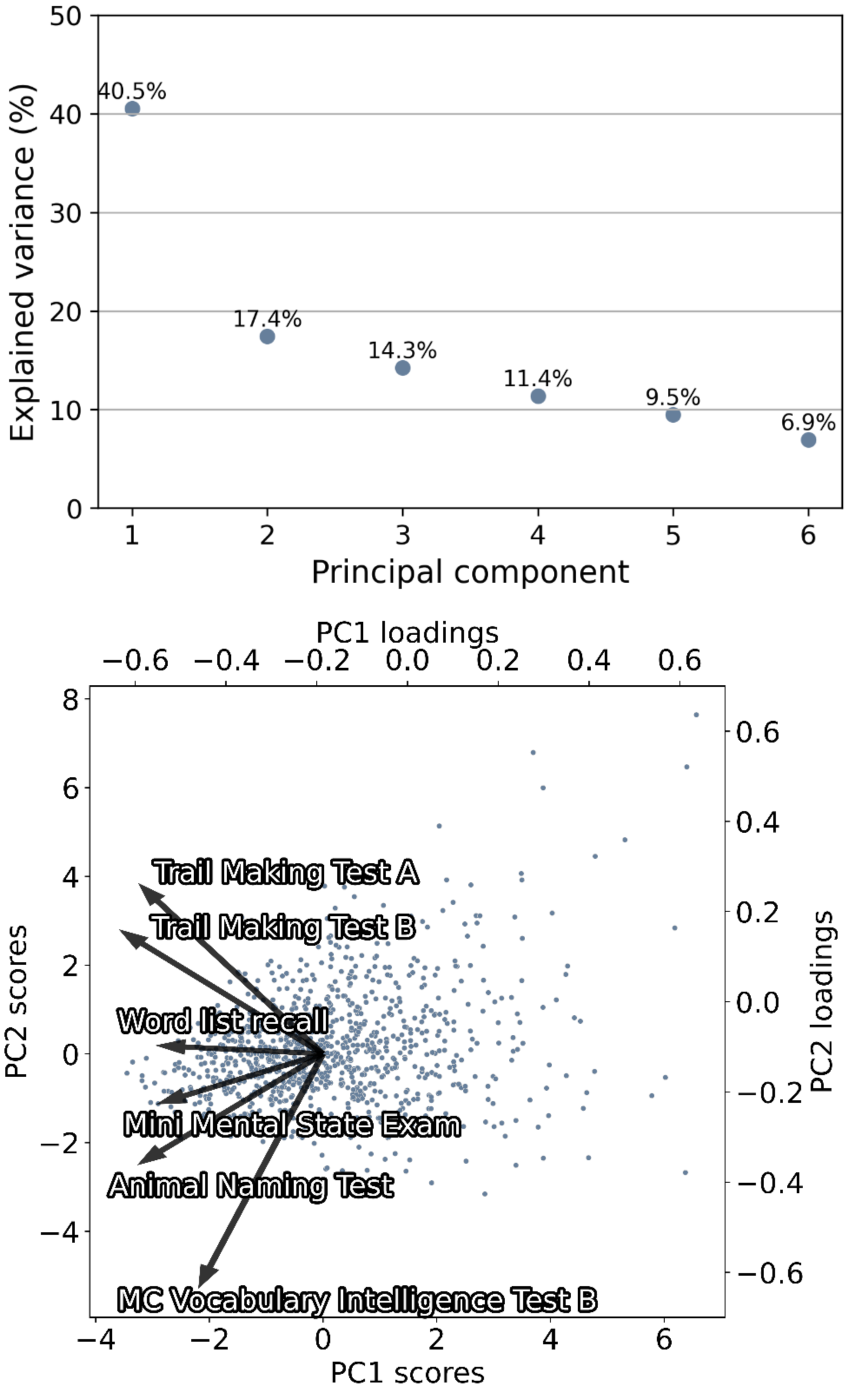

The scree plot displays the explained variance of derived principal components. The biplot displays the individual cognitive scores loadings on the principal components (arrows) and the scores of subjects (dots). *Abbreviations*: PC1 = principal component 1, PC2 = principal component 2.

Fig. S2

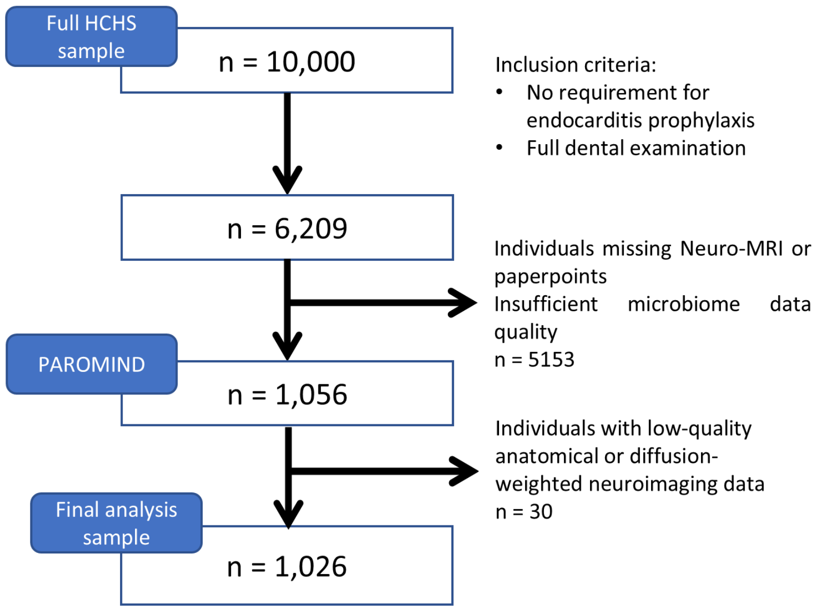

Flow chart sample selection procedure.

Fig. S3.

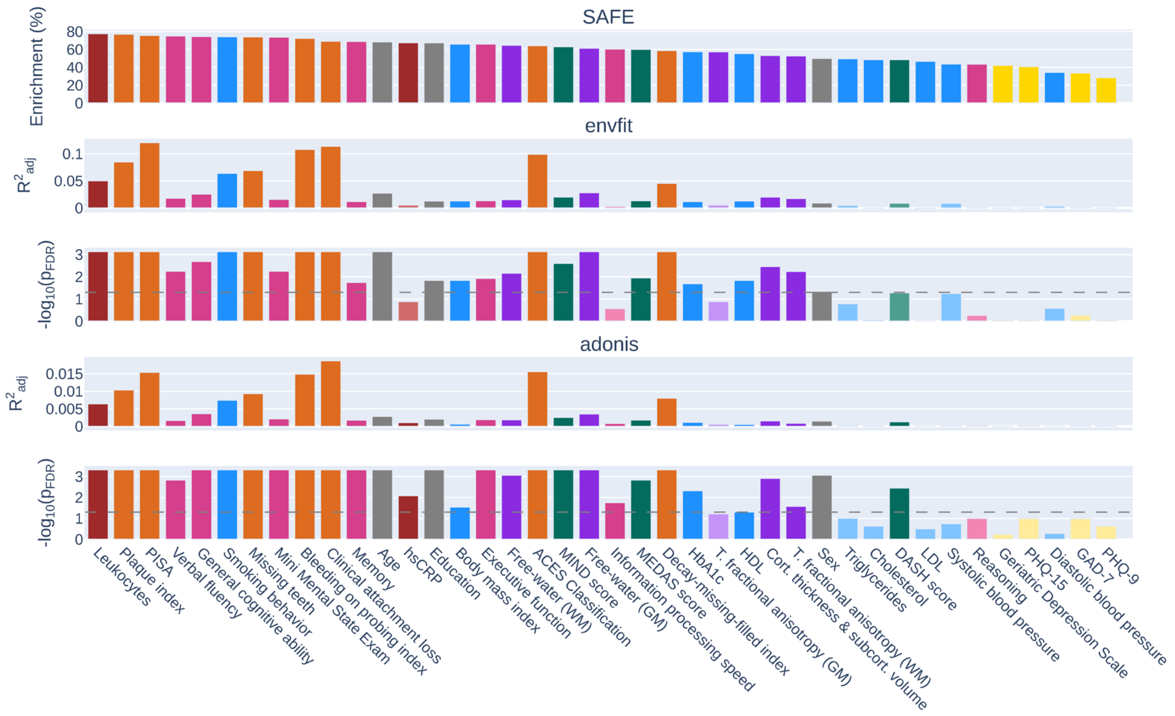

The top bar plot displays the SAFE enrichment ratio (% enrichment) for non-microbiome phenotypes, reflecting how strongly each phenotype's variance is captured by the topology of the microbiota similarity network (obtained using Aitchison distance PCoA as input), as shown in the main manuscript. As complementary approaches, we assessed the association between the same phenotypes and the overall microbiota composition (genus-level Aitchison distance matrix) using two complementary statistical methods: 1) envfit, testing the linear association between each phenotype and the PCoA ordination (all components) of the Aitchison distance matrix; 2) adonis (PERMANOVA), testing the proportion of variance in the Aitchison distance matrix explained by each phenotype individually. For both methods, we show the effect size, quantified as the adjusted R^2, and the corresponding statistical significance, displayed as $-\log10(p_{FDR})$. For all significance plots, the horizontal dashed line indicates the significance threshold of $p_{FDR} = 0.05$. The bars in all plots are colored by variable category.

Summary: Phenotypes exhibiting the highest enrichment ratios (top plot) generally show significant associations across the complementary linear tests (envfit, adonis), confirming their relationship with the overall subgingival microbiota composition. However, perfect concordance is not observed, potentially reflecting differences between the methods in their distinct statistical approaches and sensitivities to different types of patterns. The topological analysis is designed to detect the specific "shape" of an association and can identify complex, non-linear patterns, which our study found for certain phenotypes (e.g., mental health scores). In contrast, adonis performs a global test of association, and envfit is most sensitive to linear trends. This difference in what each method tests (e.g., local patterns vs. global variance) could explain the lack of perfect concordance.

Abbreviations: BOP index = bleeding on probing index; db-RDA = distance-based redundancy analysis; DMFT index = decayed/missing/filled teeth index; GM = gray matter; hsCRP = high sensitivity c-reactive peptide; LDL = low density lipoprotein, PERMANOVA = Permutational multivariate analysis of variance, R^2_{adj} = Adjusted R^2; T. fractional anisotropy = tissue fractional anisotropy; WM = white matter.

Fig. S4.

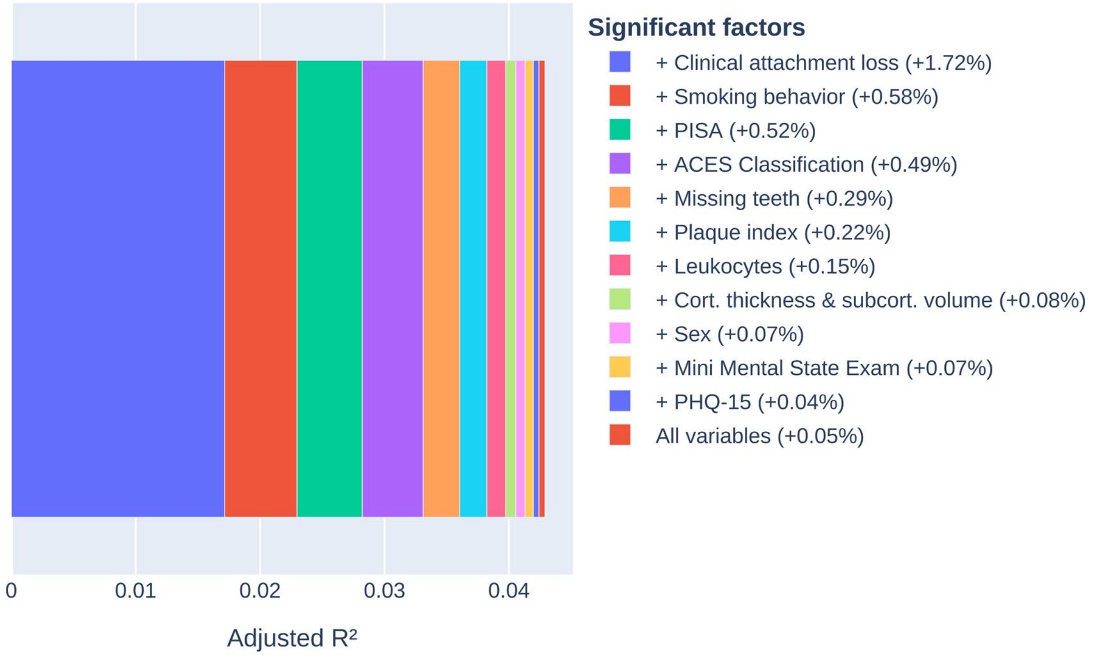

The stacked bar plot displays the cumulative variance (adjusted R^2) in the subgingival microbiota explained by significant non-microbiome phenotypes, as determined by forward model selection (ordiR2step).

Fig. S5

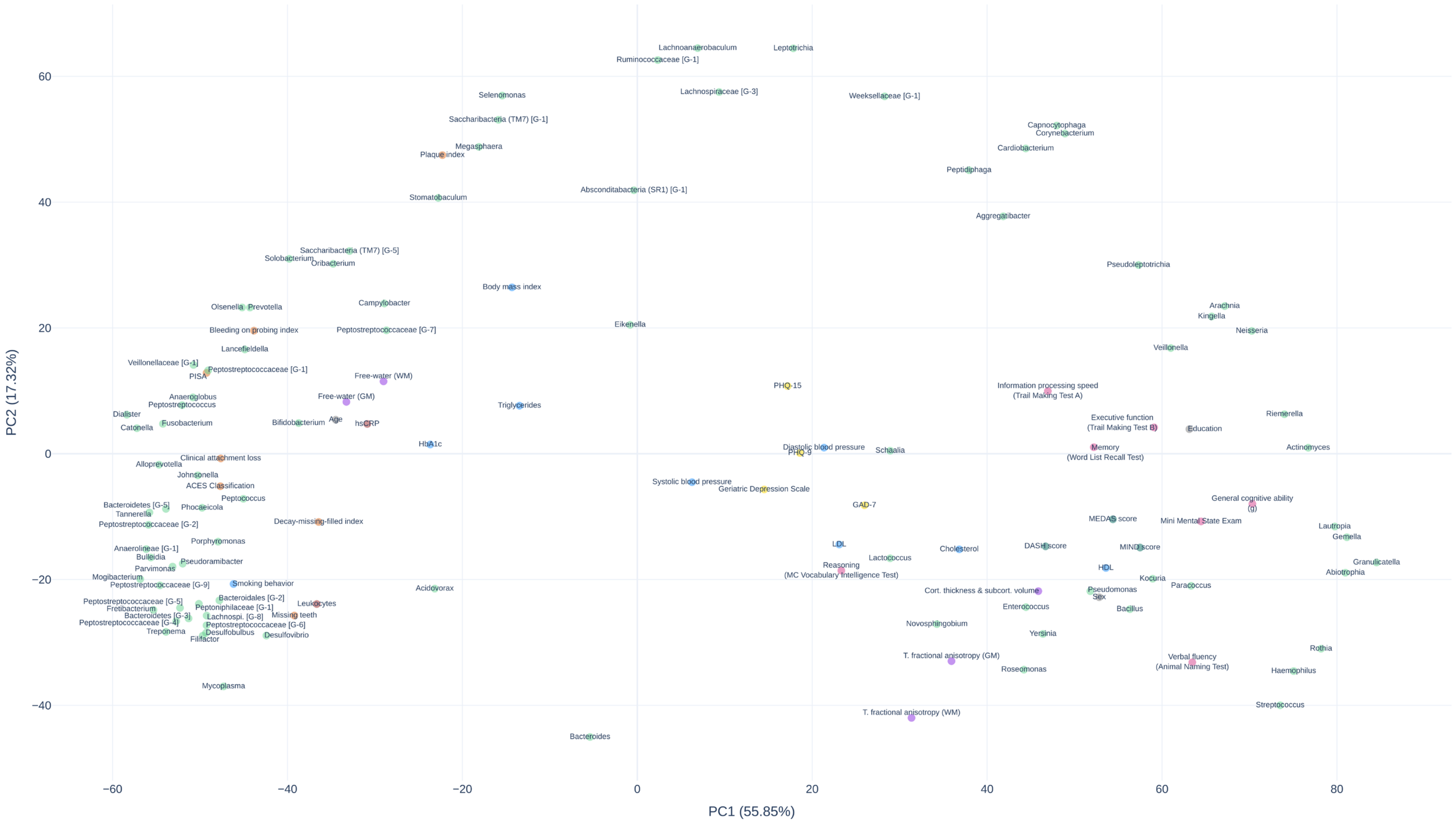

Principal component analysis of enrichment scores. The plot displays phenotypes in principal component space, where proximity indicates similar enrichment patterns. Points are color-coded by phenotype category. This is the fully annotated version of *Figure 5* in the main manuscript.

Fig. S6

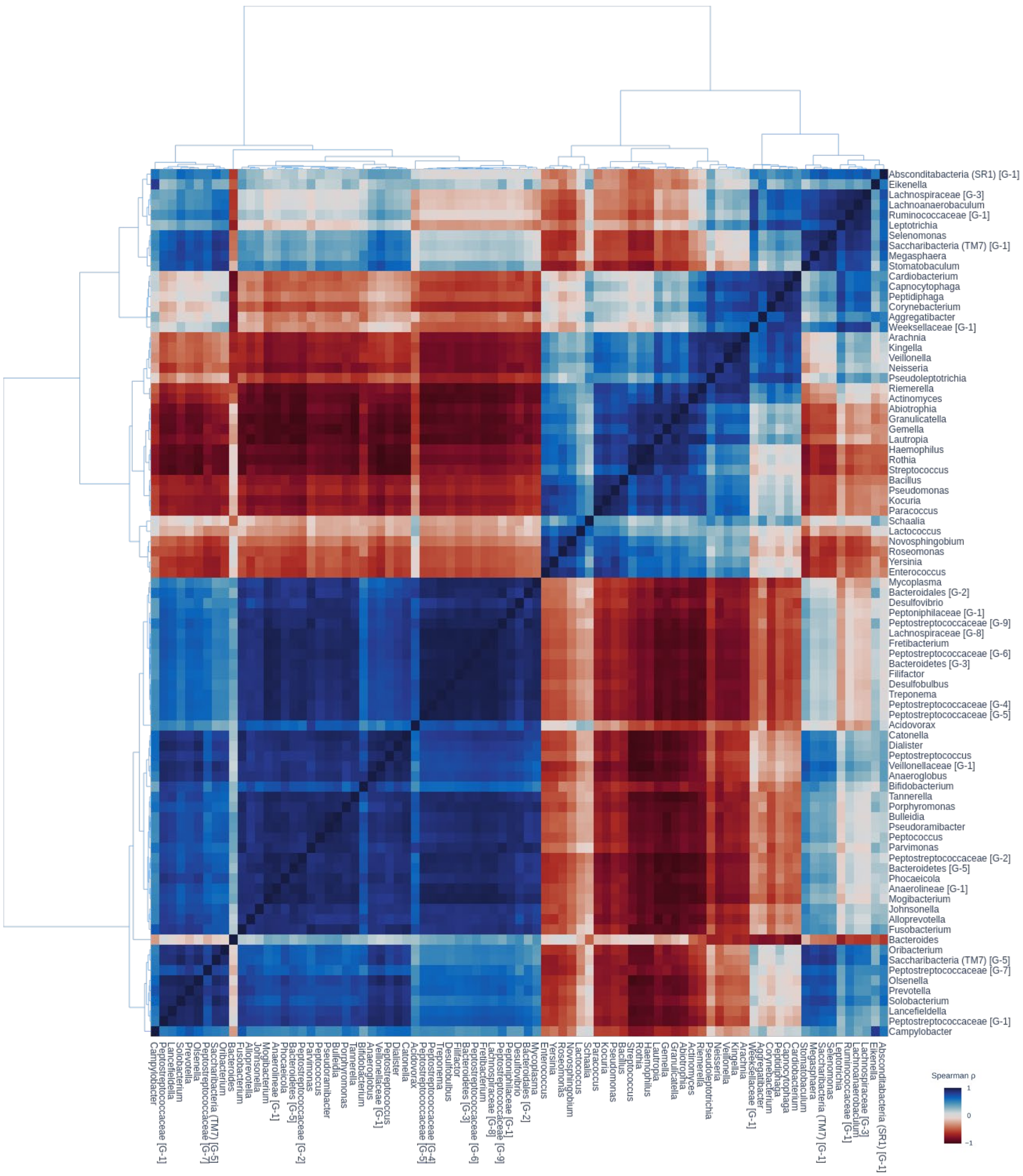

The heatmap displays the Spearman correlations of enrichment scores among bacterial genera. The genera are ordered based on hierarchical clustering of the Spearman correlations. The corresponding tree of hierarchical clustering is visualized next to the heatmap.

Fig. S7

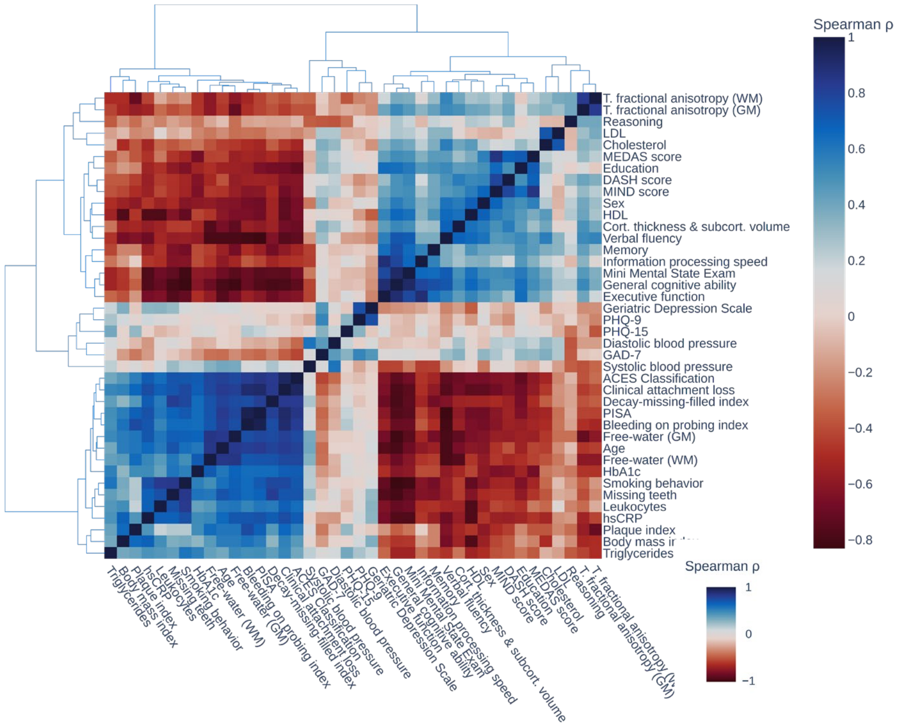

The heatmap displays the Spearman correlations of enrichment scores among non-microbiome phenotypes. The phenotypes are ordered based on hierarchical clustering of the Spearman correlations. The corresponding tree of hierarchical clustering is visualized next to the heatmap.

Fig. S8

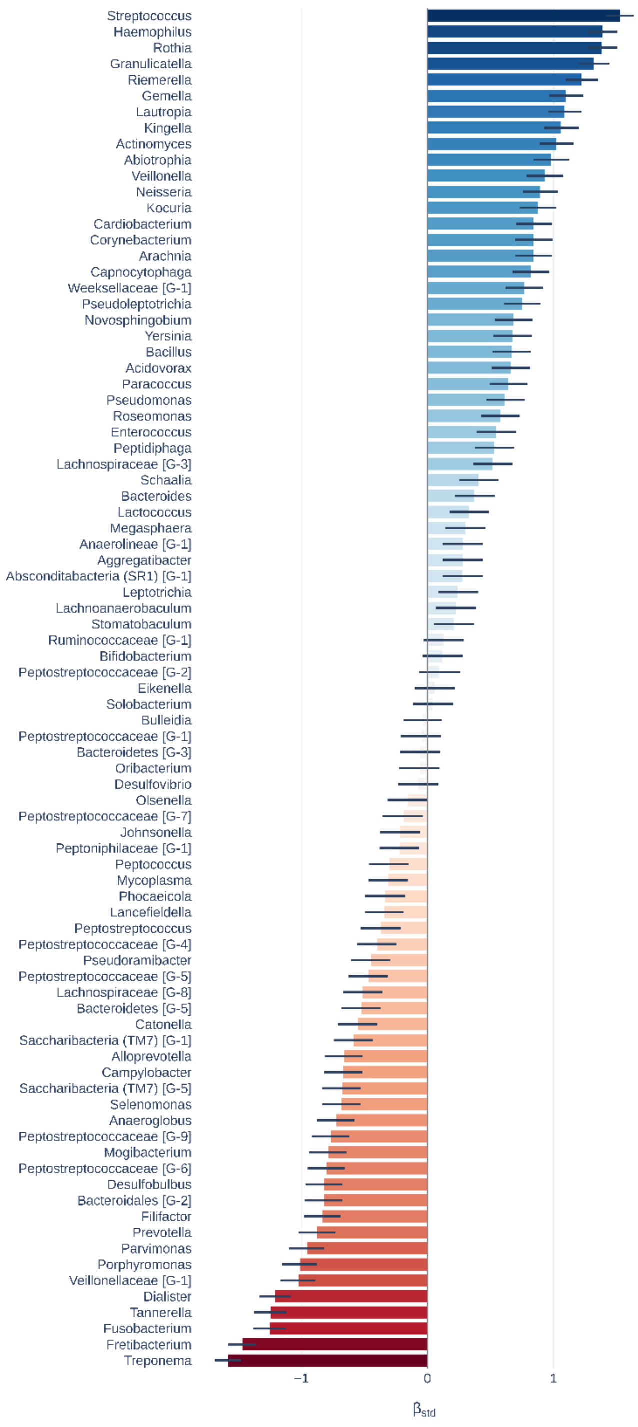

Barplot displays regression coefficients representing effects of group. Negative coefficients corresponding with higher abundance in the “A” group are shown in red, while positive coefficients indicating higher abundance in the “B” group are shown in blue. Error bars represent 95% confidence intervals.

Fig. S9

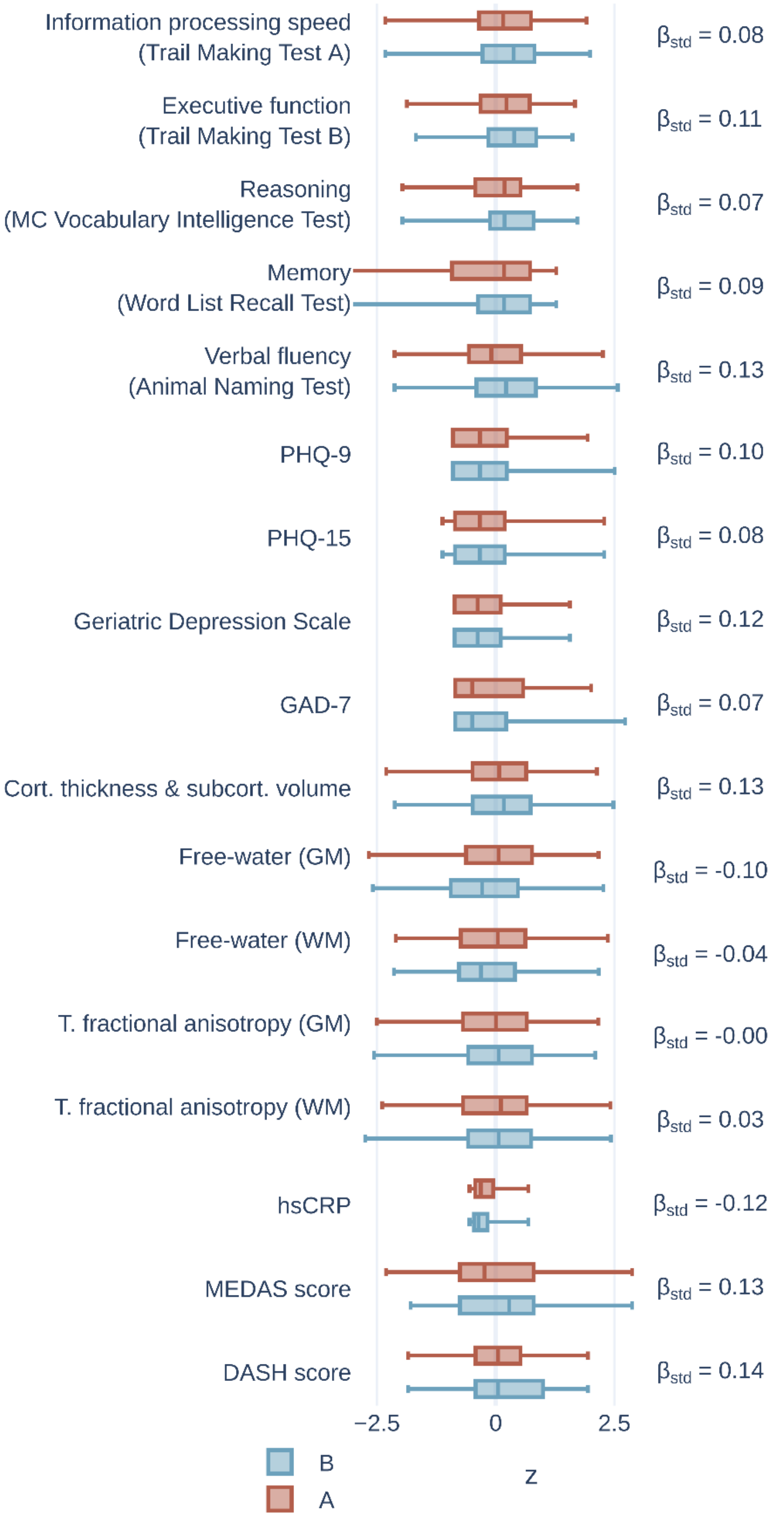

Box plot colors correspond to groups.

Fig. S10

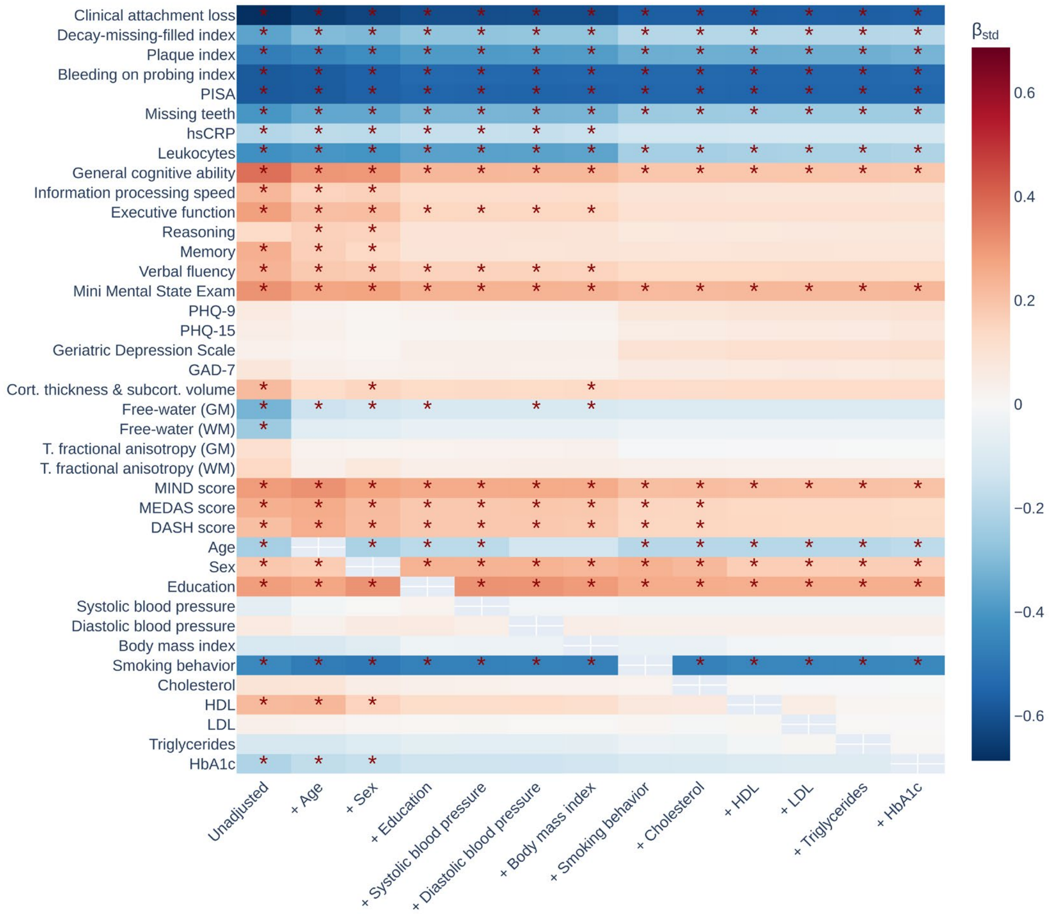

The heatmap illustrates the influence of sequential covariate adjustment on the association between microbiota group assignment and non-microbiome phenotypes. Each row represents a different phenotype, and columns represent the model at each step of adjustment, from unadjusted to fully adjusted. The color of each cell indicates the standardized beta coefficient (β_{std}) for the microbiota group variable, while red asterisks denote that the association remained statistically significant ($p < 0.05$).

Tables

Table S1. Sample characteristics of groups derived from microbiome similarity network

Metric	Group A[a] (470)	Group B[a] (310)
Age	64.49 ± 7.81 (470)	62.62 ± 8.50 (310)
Sex	38.30%	47.42%
ISCED	2.33 ± 0.62 (460)	2.50 ± 0.53 (301)
Clinical attachment loss	2.88 ± 1.04 (470)	2.32 ± 0.57 (310)
DMFT index	20.01 ± 5.13 (470)	18.22 ± 4.69 (310)
Plaque index	14.58 [0,41.1] (465)	2.17 [0, 14.8] (306)
Bleeding on probing index	13.25 [5.8, 28.6] (464)	4 [0, 11.5] (305)
Periodontal inflamed surface area	156.21 [59.9, 347.0] (464)	44.4 [0, 123.3] (305)
Missing teeth	3 [1, 8] (470)	2 [0, 5] (310)
hsCRP	0.13 [0.07, 0.29] (457)	0.1 [0.05, 0.21] (300)
Leukocytes	6.48 ± 1.81 (461)	5.74 ± 1.57 (306)
Reasoning	31.20 ± 3.54 (381)	31.61 ± 3.42 (261)
Memory	7.51 ± 1.83 (430)	7.96 ± 1.90 (297)
Verbal fluency	24.00 ± 6.30 (446)	25.52 ± 6.65 (299)
Processing speed	41.13 ± 15.03 (423)	37.64 ± 11.89 (281)
Executive function	93.40 ± 40.13 (421)	83.08 ± 33.12 (278)
Mini Mental State Exam	27.64 ± 1.68 (443)	28.14 ± 1.58 (299)
PHQ-9	2 [0, 5] (469)	2 [0, 5] (310)
PHQ-15	4.20 ± 3.77 (469)	4.38 ± 3.90 (310)
Geriatric Depression Scale	1 [0, 2] (419)	1 [0, 2] (280)
GAD-7	1 [0, 4] (469)	2 [0, 4] (310)
Systolic blood pressure	143.16 ± 20.55 (458)	141.82 ± 19.38 (303)
Diastolic blood pressure	83.24 ± 11.02 (458)	83.94 ± 10.03 (303)
MIND score	6.09 ± 1.79 (440)	6.62 ± 1.82 (295)
MEDAS score	4.26 ± 1.87 (440)	4.74 ± 1.99 (295)
DASH score	4.36 ± 1.03 (440)	4.58 ± 1.09 (295)
Body mass index	26.67 ± 4.28 (454)	26.22 ± 4.40 (304)
Smoking	24.26%	7.42%
Cholesterol	206.14 ± 40.44 (462)	209.94 ± 41.43 (305)
HDL	62.27 ± 18.34 (462)	66.70 ± 20.42 (305)
LDL	120.45 ± 37.15 (458)	121.59 ± 36.40 (302)
Triglycerides	118.77 ± 71.20 (462)	110.31 ± 78.53 (304)
HbA1c	5.68 ± 0.58 (462)	5.57 ± 0.44 (306)
Mean cortical thickness, mm	2.62 ± 0.11 (451)	2.64 ± 0.10 (298)
Mean subcortical volume, ml	3166.21 ± 307.40 (451)	3207.59 ± 314.06 (298)
Tissue fractional anisotropy (gray matter)	0.19 ± 0.01 (414)	0.19 ± 0.01 (278)

Tissue fractional anisotropy (white matter)	0.41 ± 0.01 (414)	0.41 ± 0.01 (278)
Free-water (gray matter)	0.51 ± 0.05 (414)	0.49 ± 0.05 (278)
Free-water (white matter)	0.31 ± 0.03 (414)	0.31 ± 0.02 (278)

[a]Continuous data are presented as mean ± standard deviation (SD) or, for skewed distributions, as median and interquartile range [IQR]. Categorical data are presented as percentages (%). The number of available observations (N) is indicated for each variable.

Table S2. Sample characteristics of participants based on Mapper retention

Metric	Mapper excluded[a] (109)	Mapper retained[a] (917)	p_{FDR}[b]
Age	62.28 ± 8.31 (109)	63.89 ± 8.14 (917)	0.28
Sex	45.87%	42.31%	0.881
ISCED	2.50 ± 0.54 (105)	2.41 ± 0.59 (894)	0.476
Clinical attachment loss	2.45 ± 0.68 (109)	2.62 ± 0.90 (916)	0.28
DMFT index	19.64 ± 5.40 (109)	19.22 ± 4.97 (917)	0.817
Plaque index	0 [0,17.3] (108)	8.5 [0, 30.4] (906)	0.096
Bleeding on probing index	3.57 [0, 14.0] (107)	8.92 [2.27, 20.1] (904)	<0.001**
Periodontal inflamed surface area	28.77 [0, 149.2] (107)	98.56 [25.7, 238.1] (903)	<0.001**
Missing teeth	3 [1, 7] (109)	3 [1, 6] (916)	0.988
hsCRP	0.1 [0.06, 0.21] (105)	0.12 [0.06, 0.25] (892)	0.9
Leukocytes	5.85 ± 1.92 (104)	6.16 ± 1.72 (902)	0.476
Reasoning (Multiple Choice Vocabulary Intelligence Test)	31.62 ± 2.94 (84)	31.40 ± 3.48 (754)	0.881
Memory (Word List Recall)	7.96 ± 1.93 (100)	7.67 ± 1.87 (849)	0.476
Verbal fluency (Animal Naming Test)	25.13 ± 6.61 (103)	24.60 ± 6.68 (874)	0.817
Processing speed (Trail Making Test A)	38.01 ± 12.57 (96)	40.03 ± 14.23 (829)	0.476
Executive function (Trail Making Test B)	88.82 ± 36.39 (95)	89.51 ± 37.82 (822)	0.967
Mini Mental State Exam	28.13 ± 1.46 (104)	27.82 ± 1.70 (869)	0.26
PHQ-9	2 [0, 5] (109)	2 [0, 5] (916)	0.92
PHQ-15	4.35 ± 4.12 (109)	4.27 ± 3.87 (916)	0.967
Geriatric Depression Scale	1 [0, 3] (89)	1 [0, 3] (825)	0.92
GAD-7	1 [0, 4] (109)	1 [0,4] (916)	0.741
Systolic blood pressure	140.13 ± 21.52 (106)	142.36 ± 19.88 (891)	0.724
Diastolic blood pressure	83.43 ± 10.67 (106)	83.47 ± 10.54 (891)	0.988
MIND score	6.41 ± 1.60 (106)	6.28 ± 1.80 (864)	0.817
MEDAS score	4.50 ± 1.75 (106)	4.45 ± 1.91 (864)	0.956
DASH score	4.47 ± 1.03 (106)	4.46 ± 1.06 (864)	0.988
Body mass index	26.72 ± 3.84 (106)	26.59 ± 4.37 (889)	0.92
Smoking	14.68%	17.12%	0.881
Cholesterol	208.59 ± 40.94 (105)	208.24 ± 40.84 (903)	0.988
HDL	61.89 ± 17.64 (105)	63.70 ± 19.15 (903)	0.726
LDL	122.65 ± 33.58 (103)	121.44 ± 36.88 (894)	0.92
Triglycerides	118.78 ± 72.08 (105)	117.54 ± 76.12 (902)	0.967
HbA1c	5.56 ± 0.46 (104)	5.63 ± 0.55 (903)	0.476

Mean cortical thickness, mm	2.64 ± 0.09 (103)	2.63 ± 0.11 (883)	0.724
Mean subcortical volume, ml	3283.78 ± 319.28 (103)	3177.17 ± 311.28 (883)	0.014*
Tissue fractional anisotropy (gray matter)	0.19 ± 0.01 (93)	0.19 ± 0.01 (816)	0.881
Tissue fractional anisotropy (white matter)	0.41 ± 0.01 (93)	0.41 ± 0.01 (816)	0.92
Free-water (gray matter)	0.50 ± 0.05 (93)	0.50 ± 0.05 (816)	0.724
Free-water (white matter)	0.31 ± 0.03 (93)	0.31 ± 0.03 (816)	0.988

[a]Continuous data are presented as mean ± standard deviation (SD) or, for skewed distributions, additionally as median and interquartile range [IQR]. Categorical data are presented as percentages (%). The number of available observations (N) is indicated for each variable.

[b]p-values were derived adjusting for demographics and cardiovascular risk factors. In case of individual covariates as dependent variables (e.g., age), models were adjusted for the remaining covariates (e.g., sex, education and vascular risk factors).

Table S3. Sample characteristics of participants retained versus excluded from the group analysis.

Metric	Group analysis excluded[a] (137)	Group analysis retained[a] (780)	p_{FDR}[b]
Age	64.74 ± 8.14 (137)	63.74 ± 8.13 (780)	0.547
Sex	44.53%	41.92%	0.858
ISCED	2.50 ± 0.56 (133)	2.39 ± 0.59 (761)	0.266
Clinical attachment loss	2.45 ± 0.67 (136)	2.66 ± 0.92 (780)	0.076
DMFT index	18.80 ± 4.57 (137)	19.30 ± 5.03 (780)	0.369
Plaque index	10.71 [0, 32.41] (135)	8.0 [0, 29.6] (771)	0.796
Bleeding on probing index	7.14 [3.6, 18.3] (135)	9.26 [2.27, 20.8] (769)	0.266
Periodontal inflamed surface area	72.50 [29.3, 191.77] (134)	103.12 [25.4, 245.5] (769)	0.209
Missing teeth	3 [1, 5] (136)	3 [1, 7] (780)	0.266
hsCRP	0.12 [0.07, 0.25] (135)	0.11 [0.06, 0.25] (757)	0.858
Leukocytes	6.00 ± 1.53 (135)	6.19 ± 1.75 (767)	0.547
Reasoning (Multiple Choice Vocabulary Intelligence Test)	31.57 ± 3.44 (112)	31.37 ± 3.49 (642)	0.858
Memory (Word List Recall)	7.51 ± 1.87 (122)	7.69 ± 1.87 (727)	0.656
Verbal fluency (Animal Naming Test)	24.59 ± 7.74 (129)	24.61 ± 6.48 (745)	0.983
Processing speed (Trail Making Test A)	41.66 ± 15.60 (125)	39.74 ± 13.96 (704)	0.547
Executive function (Trail Making Test B)	90.75 ± 38.00 (123)	89.30 ± 37.81 (699)	0.871
Mini Mental State Exam	27.69 ± 1.93 (127)	27.84 ± 1.66 (742)	0.738
PHQ-9	2 [1, 5] (137)	2 [0, 5] (779)	0.858
PHQ-15	4.20 ± 4.17 (137)	4.28 ± 3.82 (779)	0.948
Geriatric Depression Scale	1 [0, 3] (126)	1 [0, 2] (699)	0.266
GAD-7	1 [0, 3] (137)	2 [0, 4] (779)	0.983
Systolic blood pressure	140.83 ± 18.60 (130)	142.62 ± 20.09 (761)	0.656
Diastolic blood pressure	83.18 ± 9.97 (130)	83.52 ± 10.64 (761)	0.871
MIND score	6.18 ± 1.65 (129)	6.30 ± 1.82 (735)	0.796
MEDAS score	4.45 ± 1.76 (129)	4.45 ± 1.93 (735)	0.983
DASH score	4.55 ± 1.08 (129)	4.45 ± 1.06 (735)	0.656
Body mass index	27.16 ± 4.57 (131)	26.49 ± 4.33 (758)	0.428
Smoking	14.60%	17.56%	0.796

Cholesterol	211.59 ± 40.77 (136)	207.65 ± 40.85 (767)	0.656
HDL	61.81 ± 18.25 (136)	64.03 ± 19.30 (767)	0.547
LDL	124.48 ± 37.11 (134)	120.90 ± 36.83 (760)	0.656
Triglycerides	129.51 ± 85.15 (136)	115.41 ± 74.26 (766)	0.292
HbA1c	5.60 ± 0.63 (135)	5.64 ± 0.53 (768)	0.858
Mean cortical thickness, mm	2.63 ± 0.11 (134)	2.63 ± 0.11 (749)	0.983
Mean subcortical volume, ml	3146.42 ± 314.92 (134)	3182.67 ± 310.52 (749)	0.568
Tissue fractional anisotropy (gray matter)	0.19 ± 0.01 (124)	0.19 ± 0.01 (692)	0.871
Tissue fractional anisotropy (white matter)	0.41 ± 0.01 (124)	0.41 ± 0.01 (692)	0.858
Free-water (gray matter)	0.51 ± 0.05 (124)	0.50 ± 0.05 (692)	0.858
Free-water (white matter)	0.31 ± 0.03 (124)	0.31 ± 0.02 (692)	0.858

[a]Continuous data are presented as mean ± standard deviation (SD) or, for skewed distributions, as median and interquartile range [IQR]. Categorical data are presented as percentages (%). The number of available observations (N) is indicated for each variable.

[b]p-values were derived adjusting for demographics and cardiovascular risk factors. In case of individual covariates as dependent variables (e.g., age), models were adjusted for the remaining covariates (e.g., sex, education and vascular risk factors).

Table S4. Bootstrap regression results for genus-level relative abundance differences between microbiota similarity groups.

Genus	Avg. boot. coef.	Observed coefficient	CI lower	CI higher	Boot. p_{FDR}
Streptococcus	1.36	1.37	1.25	1.50	0.000
Rothia	1.02	1.02	0.88	1.21	0.000
Veillonella	0.81	0.81	0.66	0.98	0.000
Fusobacterium	-1.26	-1.26	-1.38	-1.14	0.000
Haemophilus	1.16	1.16	1.01	1.31	0.000
Parvimonas	-0.87	-0.87	-0.99	-0.76	0.000
Porphyromonas	-0.96	-0.96	-1.09	-0.86	0.000
Tannerella	-1.09	-1.09	-1.21	-0.97	0.000
Campylobacter	-0.63	-0.63	-0.77	-0.48	0.000
Neisseria	0.76	0.75	0.61	0.92	0.000
Dialister	-1.01	-1.01	-1.12	-0.90	0.000
Gemella	0.93	0.93	0.77	1.09	0.000
Treponema	-1.26	-1.26	-1.37	-1.17	0.000
Bacteroidales [G-2]	-0.76	-0.76	-0.90	-0.64	0.000
Filifactor	-0.80	-0.80	-0.93	-0.69	0.000
Saccharibacteria (TM7) [G-5]	-0.67	-0.67	-0.82	-0.54	0.000
Fretibacterium	-1.21	-1.21	-1.32	-1.12	0.000
Peptostreptococcaceae [G-9]	-0.85	-0.85	-0.97	-0.74	0.000
Kingella	0.76	0.75	0.60	0.95	0.000
Leptotrichia	0.15	0.14	-0.01	0.30	0.064
Prevotella	-0.85	-0.85	-0.98	-0.72	0.000
Aggregatibacter	0.20	0.20	0.05	0.38	0.016
Capnocytophaga	0.75	0.75	0.60	0.91	0.000
Granulicatella	0.99	0.98	0.85	1.15	0.000
Desulfobulbus	-0.83	-0.83	-0.96	-0.72	0.000
Corynebacterium	0.69	0.69	0.53	0.86	0.000
Actinomyces	0.82	0.82	0.68	0.98	0.000
Enterococcus	0.41	0.41	0.26	0.61	0.000
Anaeroglobus	-0.64	-0.64	-0.77	-0.54	0.000
Peptostreptococcus	-0.52	-0.52	-0.65	-0.41	0.000
Peptidiphaga	0.17	0.17	0.03	0.32	0.020
Peptostreptococcaceae [G-6]	-0.80	-0.80	-0.91	-0.70	0.000
Arachnia	0.59	0.59	0.43	0.77	0.000
Lactococcus	0.14	0.14	0.00	0.31	0.074
Riemerella	0.87	0.86	0.72	1.04	0.000
Pseudoramibacter	-0.54	-0.54	-0.68	-0.44	0.000
Mogibacterium	-0.99	-0.98	-1.11	-0.88	0.000

Saccharibacteria (TM7) [G-1]	-0.57	-0.57	-0.71	-0.43	0.000
Peptostreptococcaceae [G-5]	-0.62	-0.62	-0.75	-0.51	0.000
Alloprevotella	-0.67	-0.67	-0.79	-0.54	0.000
Phocaeicola	-0.40	-0.40	-0.52	-0.31	0.000
Cardiobacterium	0.57	0.56	0.41	0.75	0.000
Ruminococcaceae [G-1]	-0.24	-0.24	-0.38	-0.08	0.003
Pseudoleptotrichia	0.42	0.42	0.26	0.61	0.000
Lautropia	0.75	0.75	0.60	0.96	0.000
Lachnospiraceae [G-8]	-0.69	-0.69	-0.83	-0.58	0.000
Oribacterium	-0.47	-0.47	-0.61	-0.34	0.000
Catonella	-0.83	-0.83	-0.96	-0.71	0.000
Veillonellaceae [G-1]	-0.85	-0.85	-1.03	-0.75	0.000
Roseomonas	0.27	0.27	0.13	0.50	0.000
Desulfovibrio	-0.29	-0.29	-0.46	-0.20	0.000
Bacteroidetes [G-5]	-0.56	-0.57	-0.72	-0.46	0.000
Eikenella	-0.22	-0.22	-0.38	-0.08	0.003
Schaalia	0.14	0.14	-0.02	0.31	0.089
Peptoniphilaceae [G-1]	-0.40	-0.40	-0.55	-0.30	0.000
Bifidobacterium	-0.23	-0.23	-0.38	-0.13	0.000
Selenomonas	-0.63	-0.63	-0.77	-0.49	0.000
Peptostreptococcaceae [G-4]	-0.93	-0.93	-1.05	-0.83	0.000
Paracoccus	0.31	0.31	0.16	0.48	0.000
Solobacterium	-0.52	-0.52	-0.66	-0.41	0.000
Mycoplasma	-0.54	-0.54	-0.66	-0.44	0.000
Abiotrophia	0.53	0.53	0.38	0.73	0.000
Peptostreptococcaceae [G-1]	-0.55	-0.54	-0.68	-0.42	0.000
Lachnoanaerobaculum	-0.11	-0.12	-0.26	0.04	0.144
Johnsonella	-0.45	-0.45	-0.59	-0.31	0.000
Bulleidia	-0.52	-0.52	-0.65	-0.42	0.000
Stomatobaculum	-0.25	-0.26	-0.39	-0.13	0.000
Peptococcus	-0.58	-0.58	-0.72	-0.46	0.000
Pseudomonas	0.35	0.35	0.20	0.55	0.000
Lancefieldella	-0.43	-0.43	-0.56	-0.32	0.000
Peptostreptococcaceae [G-7]	-0.39	-0.39	-0.53	-0.24	0.000
Absconditabacteria (SR1) [G-1]	-0.14	-0.14	-0.29	-0.02	0.036
Bacillus	0.27	0.27	0.15	0.52	0.000
Yersinia	0.26	0.26	0.13	0.44	0.000
Olsenella	-0.49	-0.49	-0.60	-0.39	0.000
Peptostreptococcaceae [G-2]	-0.91	-0.91	-1.04	-0.81	0.000
Lachnospiraceae [G-3]	-0.04	-0.05	-0.21	0.11	0.588

Anaerolineae [G-1]	-0.65	-0.65	-0.78	-0.54	0.000
Kocuria	0.31	0.31	0.19	0.58	0.000
Weeksellaceae [G-1]	0.15	0.15	0.01	0.31	0.051
Bacteroidetes [G-3]	-0.63	-0.63	-0.76	-0.52	0.000
Megasphaera	-0.23	-0.23	-0.37	-0.11	0.000
Acidovorax	-0.22	-0.22	-0.37	-0.07	0.005
Novosphingobium	0.28	0.28	0.14	0.55	0.000
Bacteroides	-0.09	-0.09	-0.24	0.05	0.181

Table S5. Bootstrap regression results for non-microbiome phenotype differences between microbiota similarity groups.

Phenotype	Mean bootstrap coefficient	Observed coefficient	CI lower	CI higher	Bootstrap p_{FDR}
Clinical attachment loss	-0.56	-0.56	-0.70	-0.44	0.000
Plaque index	-0.32	-0.32	-0.47	-0.17	0.001
Bleeding on probing index	-0.54	-0.54	-0.67	-0.40	0.000
DMFT index	-0.19	-0.19	-0.35	-0.04	0.040
PISA	-0.55	-0.55	-0.67	-0.43	0.000
Missing teeth	-0.24	-0.24	-0.37	-0.10	0.003
Animal Naming Test	0.13	0.13	-0.01	0.28	0.137
General cognitive function	0.19	0.19	0.05	0.31	0.018
Mini Mental State Exam	0.23	0.23	0.07	0.37	0.010
Multiple Choice Vocabulary Intelligence Test	0.07	0.07	-0.07	0.21	0.385
Trail Making Test A	0.08	0.08	-0.05	0.21	0.284
Trail Making Test B	0.11	0.11	-0.03	0.23	0.154
Word List Recall	0.09	0.09	-0.05	0.23	0.284
Cortical thickness & subcortical volume	0.13	0.13	-0.01	0.27	0.137
Free-water (gray matter)	-0.10	-0.10	-0.20	0.02	0.137
Free-water (white matter)	-0.04	-0.04	-0.15	0.08	0.545
Tissue fractional anisotropy (gray matter)	0.00	0.00	-0.15	0.14	0.982
Tissue fractional anisotropy (white matter)	0.03	0.03	-0.11	0.18	0.698
hsCRP	-0.12	-0.12	-0.25	0.00	0.137
Leukocytes	-0.21	-0.21	-0.35	-0.08	0.010
PHQ-9	0.07	0.08	-0.07	0.24	0.391
PHQ-15	0.07	0.08	-0.07	0.24	0.391
Geriatric Depression Scale	0.12	0.12	-0.02	0.26	0.165
GAD-7	0.07	0.07	-0.08	0.22	0.404
MEDAS score	0.14	0.13	-0.02	0.28	0.137
DASH score	0.14	0.14	-0.01	0.28	0.137
MIND score	0.20	0.20	0.06	0.35	0.025

Table S6. Sensitivity analysis of pipeline parameter variations on enrichment ratios and group assignments.

Pipeline design variation	Spearman correlation of enrichment ratio	Adjusted Rand Index of k-Means clustering
Mapper cover overlap: 1 [default: 1.5]	0.80	0.81
Mapper cover overlap: 1.2 [default: 1.5]	0.82	0.81
Mapper cover overlap: 1.4 [default: 1.5]	0.81	0.88
Mapper cover overlap: 1.6 [default: 1.5]	0.79	0.91
Mapper cover overlap: 1.8 [default: 1.5]	0.84	0.91
Mapper cover overlap: 2 [default: 1.5]	0.83	0.90
Mapper cover resolution: 20 [default: 30]	0.88	0.72
Mapper cover resolution: 25 [default: 30]	0.85	0.81
Mapper cover resolution: 35 [default: 30]	0.82	0.87
Mapper cover resolution: 40 [default: 30]	0.85	0.88
Mapper cover resolution: 45 [default: 30]	0.82	0.87
Mapper epsilon threshold for HDBSCAN: 0.90 [default: 0.95]	0.77	0.83
Mapper epsilon threshold for HDBSCAN: 0.99 [default: 0.95]	0.83	0.76
SAFE distance threshold: 0.5 [default: 0.75]	0.85	- *
SAFE distance threshold: 0.99 [default: 0.75]	0.85	- *
SAFE neighborhood radius: 0.05 [default: 0.1]	0.84	- *
SAFE neighborhood radius: 0.15 [default: 0.1]	0.77	- *
Mean ± SD	0.82 ± 0.03	0.84 ± 0.06

This table displays sensitivity analysis results. To evaluate the potential influence of pipeline design choices on our findings, we reanalyzed the data using 17 different pipeline configurations, varying the components and parameters of the topological data analysis.

Abbreviations: HDBSCAN = Hierarchical Density-Based Spatial Clustering of Applications with Noise, SAFE = Spatial Analysis of Functional Enrichment

* The pipeline adjustments involving SAFE do not alter the k-Means clustering of the topological network as they are not relevant for the computation.

References

[1] Liao T, Wei Y, Luo M, Zhao G-P, Zhou H. tmap: an integrative framework based on topological data analysis for population-scale microbiome stratification and association studies. Genome Biol 2019;20:293. https://doi.org/10.1186/s13059-019-1871-4.

[2] Baryshnikova A. Systematic Functional Annotation and Visualization of Biological Networks. Cell Systems 2016;2:412–21. https://doi.org/10.1016/j.cels.2016.04.014.

[3] Muyzer G, de Waal EC, Uitterlinden AG. Profiling of complex microbial populations by denaturing gradient gel electrophoresis analysis of polymerase chain reaction-amplified genes coding for 16S rRNA. Appl Environ Microbiol 1993;59:695–700. https://doi.org/10.1128/aem.59.3.695-700.1993.

[4] Caporaso JG, Lauber CL, Walters WA, Berg-Lyons D, Huntley J, Fierer N, et al. Ultra-high-throughput microbial community analysis on the Illumina HiSeq and MiSeq platforms. ISME J 2012;6:1621–4. https://doi.org/10.1038/ismej.2012.8.

[5] Eke PI, Page RC, Wei L, Thornton-Evans G, Genco RJ. Update of the case definitions for population-based surveillance of periodontitis. J Periodontol 2012;83:1449–54. https://doi.org/10.1902/jop.2012.110664.

[6] Holtfreter B, Albandar JM, Dietrich T, Dye BA, Eaton KA, Eke PI, et al. Standards for reporting chronic periodontitis prevalence and severity in epidemiologic studies: Proposed standards from the Joint EU/USA Periodontal Epidemiology Working Group. J Clin Periodontol 2015;42:407–12. https://doi.org/10.1111/jcpe.12392.

[7] Papapanou PN, Sanz M, Buduneli N, Dietrich T, Feres M, Fine DH, et al. Periodontitis: Consensus report of workgroup 2 of the 2017 World Workshop on the Classification of Periodontal and Peri-Implant Diseases and Conditions. Journal of Clinical Periodontology 2018;45:S162–70. https://doi.org/10.1111/jcpe.12946.

[8] Caton JG, Armitage G, Berglundh T, Chapple ILC, Jepsen S, Kornman KS, et al. A new classification scheme for periodontal and peri-implant diseases and conditions – Introduction and key changes from the 1999 classification. Journal of Clinical Periodontology 2018;45:S1–8. https://doi.org/10.1111/jcpe.12935.

[9] Holtfreter B, Kuhr K, Borof K, Tonetti MS, Sanz M, Kornman K, et al. ACES: A new framework for the application of the 2018 periodontal status classification scheme to epidemiological survey data. J Clin Periodontol 2024;51:512–21. https://doi.org/10.1111/jcpe.13965.

[10] Moms JC, Heyman A, Mohs RC, Hughes JP, van Belle G, Fillenbaum G, et al. The Consortium to Establish a Registry for Alzheimer's Disease (CERAD). Part I. Clinical and neuropsychological assesment of Alzheimer's disease. Neurology 1989;39:1159–1159. https://doi.org/10.1212/WNL.39.9.1159.

[11] Fawns-Ritchie C, Deary IJ. Reliability and validity of the UK Biobank cognitive tests. PLoS One 2020;15:e0231627. https://doi.org/10.1371/journal.pone.0231627.

[12] Kroenke K, Spitzer RL, Williams JBW. The PHQ-9: Validity of a brief depression severity measure. J Gen Intern Med 2001;16:606–13. https://doi.org/10.1046/j.1525-1497.2001.016009606.x.

[13] Kroenke K, Spitzer RL, Williams JBW. The PHQ-15: Validity of a New Measure for Evaluating the Severity of Somatic Symptoms: Psychosomatic Medicine 2002;64:258–66. https://doi.org/10.1097/00006842-200203000-00008.

[14] Spitzer RL, Kroenke K, Williams JBW, Löwe B. A Brief Measure for Assessing Generalized Anxiety Disorder: The GAD-7. Arch Intern Med 2006;166:1092. https://doi.org/10.1001/archinte.166.10.1092.

[15] Petersen M, Nägele FL, Mayer C, Schell M, Petersen E, Kühn S, et al. Brain imaging and neuropsychological assessment of individuals recovered from a mild to moderate SARS-CoV-2 infection. Proceedings of the National Academy of Sciences 2023;120:e2217232120. https://doi.org/10.1073/pnas.2217232120.
[16] Dale AM, Fischl B, Sereno MI. Cortical surface-based analysis. I. Segmentation and surface reconstruction. Neuroimage 1999;9:179–94. https://doi.org/10.1006/nimg.1998.0395.
[17] Pasternak O, Sochen N, Gur Y, Intrator N, Assaf Y. Free water elimination and mapping from diffusion MRI. Magnetic Resonance in Medicine 2009;62:717–30. https://doi.org/10.1002/mrm.22055.
[18] Cieslak M, Cook PA, He X, Yeh F-C, Dhollander T, Adebimpe A, et al. QSIPrep: an integrative platform for preprocessing and reconstructing diffusion MRI data. Nat Methods 2021;18:775–8. https://doi.org/10.1038/s41592-021-01185-5.
[19] Veraart J, Novikov DS, Christiaens D, Ades-aron B, Sijbers J, Fieremans E. Denoising of diffusion MRI using random matrix theory. Neuroimage 2016;142:394–406. https://doi.org/10.1016/j.neuroimage.2016.08.016.
[20] Kellner E, Dhital B, Kiselev VG, Reisert M. Gibbs-ringing artifact removal based on local subvoxel-shifts. Magn Reson Med 2016;76:1574–81. https://doi.org/10.1002/mrm.26054.
[21] Andersson JLR, Sotiropoulos SN. An integrated approach to correction for off-resonance effects and subject movement in diffusion MR imaging. Neuroimage 2016;125:1063–78. https://doi.org/10.1016/j.neuroimage.2015.10.019.
[22] Tustison NJ, Avants BB, Cook PA, Zheng Y, Egan A, Yushkevich PA, et al. N4ITK: improved N3 bias correction. IEEE Trans Med Imaging 2010;29:1310–20. https://doi.org/10.1109/TMI.2010.2046908.
[23] Esteban O, Birman D, Schaer M, Koyejo OO, Poldrack RA, Gorgolewski KJ. MRIQC: Advancing the automatic prediction of image quality in MRI from unseen sites. PLOS ONE 2017;12:e0184661. https://doi.org/10.1371/journal.pone.0184661.
[24] Fischl B, Salat DH, Busa E, Albert M, Dieterich M, Haselgrove C, et al. Whole brain segmentation: automated labeling of neuroanatomical structures in the human brain. Neuron 2002;33:341–55. https://doi.org/10.1016/s0896-6273(02)00569-x.
[25] Fischl B, Dale AM. Measuring the thickness of the human cerebral cortex from magnetic resonance images. Proceedings of the National Academy of Sciences 2000;97:11050–5. https://doi.org/10.1073/pnas.200033797.
[26] Pasternak O, Sochen N, Gur Y, Intrator N, Assaf Y. Free water elimination and mapping from diffusion MRI. Magnetic Resonance in Medicine 2009;62:717–30. https://doi.org/10.1002/mrm.22055.
[27] Boeing H, Wahrendorf J, Becker N. EPIC-Germany--A source for studies into diet and risk of chronic diseases. European Investigation into Cancer and Nutrition. Ann Nutr Metab 1999;43:195–204. https://doi.org/10.1159/000012786.
[28] Hebestreit K, Yahiaoui-Doktor M, Engel C, Vetter W, Siniatchkin M, Erickson N, et al. Validation of the German version of the Mediterranean Diet Adherence Screener (MEDAS) questionnaire. BMC Cancer 2017;17:341. https://doi.org/10.1186/s12885-017-3337-y.
[29] Folsom AR, Parker ED, Harnack LJ. Degree of Concordance With DASH Diet Guidelines and Incidence of Hypertension and Fatal Cardiovascular Disease*: American Journal of Hypertension 2007;20:225–32. https://doi.org/10.1016/j.amjhyper.2006.09.003.

[30] Morris MC, Tangney CC, Wang Y, Sacks FM, Barnes LL, Bennett DA, et al. MIND diet slows cognitive decline with aging. Alzheimer's & Dementia 2015;11:1015–22. https://doi.org/10.1016/j.jalz.2015.04.011.
[31] Saggar M, Sporns O, Gonzalez-Castillo J, Bandettini PA, Carlsson G, Glover G, et al. Towards a new approach to reveal dynamical organization of the brain using topological data analysis. Nat Commun 2018;9:1399. https://doi.org/10.1038/s41467-018-03664-4.
[32] Saggar M, Shine JM, Liégeois R, Dosenbach NUF, Fair D. Precision dynamical mapping using topological data analysis reveals a hub-like transition state at rest. Nat Commun 2022;13:4791. https://doi.org/10.1038/s41467-022-32381-2.
[33] Nicolau M, Levine AJ, Carlsson G. Topology based data analysis identifies a subgroup of breast cancers with a unique mutational profile and excellent survival. Proc Natl Acad Sci U S A 2011;108:7265–70. https://doi.org/10.1073/pnas.1102826108.
[34] Yao Y, Sun J, Huang X, Bowman GR, Singh G, Lesnick M, et al. Topological methods for exploring low-density states in biomolecular folding pathways. J Chem Phys 2009;130:144115. https://doi.org/10.1063/1.3103496.
[35] Romano D, Nicolau M, Quintin E-M, Mazaika PK, Lightbody AA, Cody Hazlett H, et al. Topological methods reveal high and low functioning neuro-phenotypes within fragile X syndrome. Hum Brain Mapp 2014;35:4904–15. https://doi.org/10.1002/hbm.22521.
[36] Singh G, Memoli F, Ishkhanov T, Sapiro G, Carlsson G, Ringach DL. Topological analysis of population activity in visual cortex. Journal of Vision 2008;8:11. https://doi.org/10.1167/8.8.11.
[37] Singh G, Memoli F, Carlsson G. Topological Methods for the Analysis of High Dimensional Data Sets and 3D Object Recognition. The Eurographics Association; 2007.
[38] Gloor GB, Macklaim JM, Pawlowsky-Glahn V, Egozcue JJ. Microbiome Datasets Are Compositional: And This Is Not Optional. Front Microbiol 2017;8. https://doi.org/10.3389/fmicb.2017.02224.
[39] Martino C, Morton JT, Marotz CA, Thompson LR, Tripathi A, Knight R, et al. A Novel Sparse Compositional Technique Reveals Microbial Perturbations. mSystems 2019;4:e00016-19. https://doi.org/10.1128/mSystems.00016-19.
[40] McInnes L, Healy J, Astels S. hdbscan: Hierarchical density based clustering. Journal of Open Source Software 2017;2:205. https://doi.org/10.21105/joss.00205.
[41] Costanzo M, VanderSluis B, Koch EN, Baryshnikova A, Pons C, Tan G, et al. A global genetic interaction network maps a wiring diagram of cellular function. Science 2016;353:aaf1420. https://doi.org/10.1126/science.aaf1420.
[42] Fruchterman TMJ, Reingold EM. Graph drawing by force-directed placement. Software: Practice and Experience 1991;21:1129–64. https://doi.org/10.1002/spe.4380211102.
[43] Ikotun AM, Ezugwu AE, Abualigah L, Abuhaija B, Heming J. K-means clustering algorithms: A comprehensive review, variants analysis, and advances in the era of big data. Information Sciences 2023;622:178–210. https://doi.org/10.1016/j.ins.2022.11.139.
[44] Mayer C, Walther C, Borof K, Nägele FL, Petersen M, Schell M, et al. Association between periodontal disease and microstructural brain alterations in the Hamburg City Health Study. Journal of Clinical Periodontology 2023;n/a. https://doi.org/10.1111/jcpe.13828.

[45] Alfaro-Almagro F, McCarthy P, Afyouni S, Andersson JLR, Bastiani M, Miller KL, et al. Confound modelling in UK Biobank brain imaging. NeuroImage 2021;224:117002. https://doi.org/10.1016/j.neuroimage.2020.117002.
[46] Petersen M, Hoffstaedter F, Nägele FL, Mayer C, Schell M, Rimmele DL, et al. A latent clinical-anatomical dimension relating metabolic syndrome to brain structure and cognition. eLife 2024;12:RP93246. https://doi.org/10.7554/eLife.93246.
[47] Sun Q, Li M. Association between periodontitis and cognitive impairment in older adults: A cross-sectional study of the National Health and Nutrition Examination Survey. Clinical Epidemiology and Global Health 2025:102020. https://doi.org/10.1016/j.cegh.2025.102020.
[48] Blanchet FG, Legendre P, Borcard D. Forward Selection of Explanatory Variables. Ecology 2008;89:2623–32. https://doi.org/10.1890/07-0986.1.
